# Intestinal Inflammation Reversibly Alters the Microbiota to Drive Susceptibility to Clostridioides difficile Colonization in a Mouse Model of Colitis

**DOI:** 10.1128/mbio.01904-22

**Published:** 2022-07-28

**Authors:** Madeline R. Barron, Kelly L. Sovacool, Lisa Abernathy-Close, Kimberly C. Vendrov, Alexandra K. Standke, Ingrid L. Bergin, Patrick D. Schloss, Vincent B. Young

**Affiliations:** a Department of Microbiology & Immunology, University of Michigan Medical School, Ann Arbor, Michigan, USA; b Department of Computational Medicine & Bioinformatics, University of Michigan Medical School, Ann Arbor Michigan, USA; c Division of Rheumatology, Department of Internal Medicine, University of Michigan Medical School, Ann Arbor, Michigan, USA; d Division of Infectious Diseases, Department of Internal Medicine, University of Michigan Medical School, Ann Arbor, Michigan, USA; e The Unit for Laboratory Animal Medicine, University of Michigan Medical School, Ann Arbor, Michigan, USA; University of Oklahoma Health Sciences Center

**Keywords:** *Clostridioides difficile*, *Helicobacter hepaticus*, animal models, gut inflammation, gut microbiota, intestinal colonization

## Abstract

Susceptibility to Clostridioides difficile infection (CDI) typically follows the administration of antibiotics. Patients with inflammatory bowel disease (IBD) have increased incidence of CDI, even in the absence of antibiotic treatment. However, the mechanisms underlying this susceptibility are not well understood. To explore the intersection between CDI and IBD, we recently described a mouse model where colitis triggered by the murine gut bacterium, Helicobacter hepaticus, in IL-10^−/−^ mice led to susceptibility to C. difficile colonization without antibiotic administration. The current work disentangles the relative contributions of inflammation and gut microbiota in colonization resistance to C. difficile in this model. We show that inflammation drives changes in microbiota composition, which leads to CDI susceptibility. Decreasing inflammation with an anti-p40 monoclonal antibody promotes a shift of the microbiota back toward a colonization-resistant state. Transferring microbiota from susceptible and resistant mice to germfree animals transfers the susceptibility phenotype, supporting the primacy of the microbiota in colonization resistance. These findings shine light on the complex interactions between the host, microbiota, and C. difficile in the context of intestinal inflammation, and may form a basis for the development of strategies to prevent or treat CDI in IBD patients.

## INTRODUCTION

The mammalian gastrointestinal tract is inhabited by a diverse community of microbes that contributes to colonization resistance against pathogenic organisms, including the toxin-producing bacterium Clostridioides difficile (1, 2). Disruption of the microbiota, typically in the setting of treatment with antibiotics, allows C. difficile to establish within the gut (3–7). C. difficile colonization can lead to clinical syndromes ranging from mild diarrhea to severe colitis (8). Host immune responses also support colonization resistance to the pathogen, in part through modulating the structure and function of the gut microbial community (9–11).

The interplay between the host and microbiota also underlies the pathogenesis of the inflammatory bowel diseases (IBD) Crohn’s disease and ulcerative colitis. IBD results from dysregulated interactions between host immune responses and the microbiota in genetically susceptible individuals, leading to chronic, progressive inflammation of the gut (12). IBD patients harbor a distinct microbiota compared to healthy individuals (13). These changes in microbiota structure and associated inflammation in IBD are accompanied by an altered intestinal metabolome, characterized by decreases in short-chain fatty acid concentrations and altered bile acid profiles (14–16). Notably, patients with IBD have an increased risk of developing C. difficile infection (CDI) (17–21), even in the absence of antibiotic treatment (22). However, despite the known associations between inflammation and microbiota alterations in IBD, the mechanisms permitting colonization by C. difficile in IBD patients remain unclear.

To explore the intersection between CDI susceptibility and IBD, we developed a mouse model in which C. difficile colonization occurs in the absence of antibiotic pretreatment (10). This is in marked contrast to most models of CDI, where antibiotic administration is required to make animals susceptible to infection (4, 23, 24). In our novel system, IL-10^−/−^ mice are colonized by Helicobacter hepaticus, a murine gut bacterium that triggers colitis in genetically predisposed hosts (25, 26). The combination of intestinal inflammation, coupled with changes in microbiota composition, is sufficient to render colitic mice susceptible to colonization by C. difficile (10). However, the relative contributions of inflammation and microbiota alterations underlying susceptibility to C. difficile in this model remain to be explored.

In the current study, we sought to separate the differential role of host responses and changes to the microbiota that lead to loss of resistance to C. difficile colonization in mice with colitis. To do this, we follow up on observations that the proinflammatory cytokine IL-23 drives H. hepaticus-triggered colitis in mice with defective regulatory immune signaling (27, 28). We show that inflammation changes the composition of the gut microbiota to permit C. difficile colonization. Subsequently, treating inflammation with a monoclonal antibody that targets the p40 subunit of IL-23 restores resistance to C. difficile. Transferring the microbiota from colonization resistant or inflammation-induced susceptible IL-10^−/−^ mice to germfree wild-type mice is sufficient to transfer the susceptibility phenotype, underscoring the central role of the microbiota in colonization resistance to C. difficile. Together, our results provide insights into mechanisms by which the host-microbiota interface mediates susceptibility to C. difficile colonization in the context of intestinal inflammation.

## RESULTS

### Treatment of inflammation in colitic mice by anti-p40 monoclonal antibody inhibits susceptibility to C. difficile colonization.

In our previous work, we hypothesized that inflammation promotes susceptibility to C. difficile colonization in mice with colitis triggered by H. hepaticus (10). To further explore this hypothesis, we sought to determine whether treating established colitis would restore colonization resistance to C. difficile in this model.

**FIG 1:**
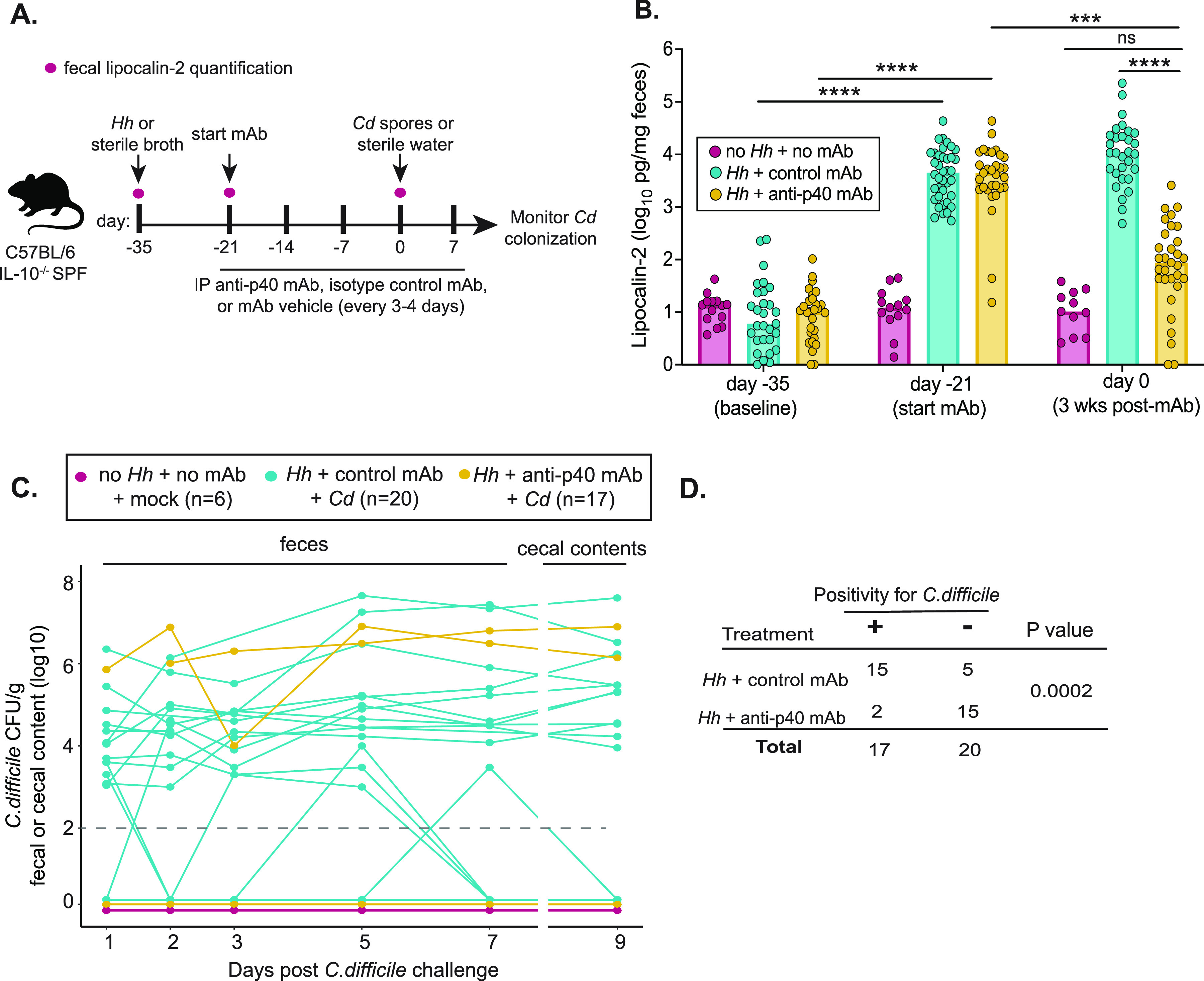
Treatment of inflammation in colitic mice by anti-p40 monoclonal antibody inhibits susceptibility to C. difficile colonization. (A) Experimental design. Mice were inoculated with H. hepaticus or sterile broth (non-IBD controls) via oral gavage. On day -21, animals were administered anti-p40 mAb or isotype control mAb via intraperitoneal (IP) injection every 3 to 4 days for 3 weeks. Non-IBD control animals received mAb vehicle. Intestinal inflammation before and after colitis development, and after 3 weeks of mAb treatment, was monitored by measuring fecal levels of lipocalin-2. Mice were then challenged with spores from C. difficile strain VPI10463 (day 0) and C. difficile colonization monitored over time. Control animals were mock challenged with sterile water. *Hh =* H. hepaticus, *Cd =* C. difficile. (B) Lipocalin-2 levels were measured via ELISA using feces collected from mice at baseline (day -35), after the development of IBD (day -21, first day of mAb treatment) and after 3 weeks of treatment with mAb or vehicle (day 0 of C. difficile challenge). Data are from 3 independent experiments. A Kruskal Wallis test followed by the Dunn’s multiple comparison’s test was performed (***, *P* < 0.001; ****, *P* < 0.0001) See also Fig. S1. (C) Shedding of C. difficile in feces collected from animals over time, and in cecal contents collected at harvest (day 9). Positivity was determined as having viable C. difficile in intestinal contents after plating on TCCFA plates. The dashed line refers to the limit of C. difficile colonization detection (10^2^ CFU/g). Each line in the graph refers to a single mouse; lines below the limit of detection include multiple mice within each group. Data are from 2 independent experiments. See also Fig. S2 and S3. (D) Fisher’s exact test of mice with IBD depicted in (C) treated with isotype control mAb or anti-p40 mAb that were positive or negative for C. difficile at any point throughout the experiments.

C57BL/6 IL-10^−/−^ SPF mice were inoculated with H. hepaticus while non-IBD control animals received sterile tryptic soy broth via oral gavage (Fig. 1A). Intestinal inflammation was monitored by measuring fecal levels of the inflammatory marker, lipocalin-2 (Fig. 1A and B). Animals colonized with H. hepaticus developed colitis, as indicated by a significant increase in fecal lipocalin-2 concentrations relative to control mice 2 weeks after inoculation with H. hepaticus (Fig. 1B; day −21). At this time, anti-p40 monoclonal antibody (mAb), an isotype control mAb, or mAb vehicle was administered to animals by intraperitoneal injection every 3 to 4 days for 3 weeks (Fig. 1A). After 3 weeks of mAb administration, there was a reduction in lipocalin-2 concentrations in mice treated with anti-p40 mAb while levels remained elevated in mice that received the isotype control mAb (Fig. 1B). Histopathologic analysis of colonic tissue collected from mice after 3 weeks of antibody treatment (Fig. S1A) supported a significant decrease in inflammation in mice treated with anti-p40 mAb (Fig. S1B). Examination of these tissues revealed reduced hyperplasia and restoration of goblet cell mass in anti-p40 mAb-treated animals relative to those treated with control mAb (Fig. S1C). Moreover, colonic expression of genes encoding the cytokines IL-17A, IFN-γ, TNF-*a*, and IL-22 was also significantly lower in mice treated with anti-p40 relative to control mAb-treated animals (Fig. S1D-G). Thus, anti-p40 mAb effectively mitigates colitis in IL-10^−/−^ animals from our breeding colony, as previously reported (27, 28).

10.1128/mbio.01904-22.1 FIG S1 Histopathological and gene expression analyses of colon tissue from IL-10^−/−^ mice without IBD and mice with IBD treated with control mAb or anti-p40 mAb. (A) Experimental timeline. Animals were inoculated with H. hepaticus *(Hh)* or sterile tryptic soy broth (non-IBD controls) via oral gavage. Fourteen days later (day 0), anti-p40 mAb, isotype control mAb, or vehicle were administered to mice via intraperitoneal (IP) injection every3 to 4 days for 3 weeks. Mice were then euthanized and colon tissue collected for histological and RT-PCR analyses. (B) Histopathological damage in the colons collected from mice without colitis and with colitis treated with control or anti-p40 mAb. The degree of lymphocytic inflammation was determined using a 4-point scale. Data from 2 independent experiments were analyzed using a Kruskal Wallis test followed by the Dunn’s multiple comparison’s test (**, *P* < 0.01; ****, *P* < 0.0001). (C) Colon tissue collected from mice colonized with H. hepaticus or mock colonized with sterile broth after 3 weeks of antibody treatment. Representative hematoxylin and eosin images are shown. (D-G) RT-PCR analysis of genes downstream of p40-mediated signaling pathways in colon tissue collected from mice with and without IBD after 3 weeks of treatment with mAb or vehicle. Results are from 2 independent experiments. Expression of β-actin was used to normalize RNA in samples. Fold change was calculated relative to a sample within the “*Hh* ^+^ control mAb” group for each gene. Statistical significance was determined via an ANOVA followed by Tukey’s test (D, E, F) or Kruskal Wallis with Dunn’s multiple comparison’s test (G) (*, *P* < 0.05; **, *P* < 0.01; ***, *P* < 0.001; ****, *P* < 0.0001). Download FIG S1, TIF file, 0.9 MB. Copyright © 2022 Barron et al. 2022 Barron et al. https://creativecommons.org/licenses/by/4.0/ This content is distributed under the terms of the Creative Commons Attribution 4.0 International license.

10.1128/mbio.01904-22.2 FIG S2 C. difficile colonization in mice treated with control mAb or anti-p40 mAb, plotted by individual animal. Shedding of C. difficile in feces collected from animals depicted in Fig. 1C over time, and in cecal contents collected at harvest (day 9). Each plot refers to a single animal. Positivity was determined as having viable C. difficile in intestinal contents after plating on TCCFA plates. The dashed line refers to the limit of C. difficile colonization detection (10^2^ CFU/g). Data are from 2 independent experiments. Download FIG S2, PDF file, 0.1 MB. Copyright © 2022 Barron et al. 2022 Barron et al. https://creativecommons.org/licenses/by/4.0/ This content is distributed under the terms of the Creative Commons Attribution 4.0 International license.

10.1128/mbio.01904-22.3 FIG S3 Clinical and histopathological scores of mice with IBD and treated with control mAb or anti-p40 mAb and challenged with C. difficile. (A) Clinical disease of mice with colitis on day 9 post challenge with C. difficile (day of sacrifice; animals are the same as those depicted in Fig. 1 in the main text). Disease severity did not significantly differ between animals that were positive or negative for C. difficile at any point throughout the experiments within each treatment group, or between groups. A Kruskal Wallis test followed by the Dunn’s multiple comparison’s test was performed. (B) Cecal and (C) colon histopathology scores (composite score of edema, epithelial damage, and inflammatory cell infiltration) in mice with IBD treated with control mAb or anti-p40 mAb and challenged with C. difficile. *Hh =* H. hepaticus*, Cd =* C. difficile. Download FIG S3, PDF file, 0.1 MB. Copyright © 2022 Barron et al. 2022 Barron et al. https://creativecommons.org/licenses/by/4.0/ This content is distributed under the terms of the Creative Commons Attribution 4.0 International license.

To test the hypothesis that treating inflammation restores resistance to C. difficile colonization, mice were challenged with either ~3 × 10^4^ spores of C. difficile strain VPI10463 or sterile water via oral gavage after 3 weeks of mAb treatment (Fig. 1A; day 0). Animals were monitored for C. difficile colonization and disease and continued to receive mAb injections throughout the rest of the experiment (Fig. 1A).

**FIG 2:**
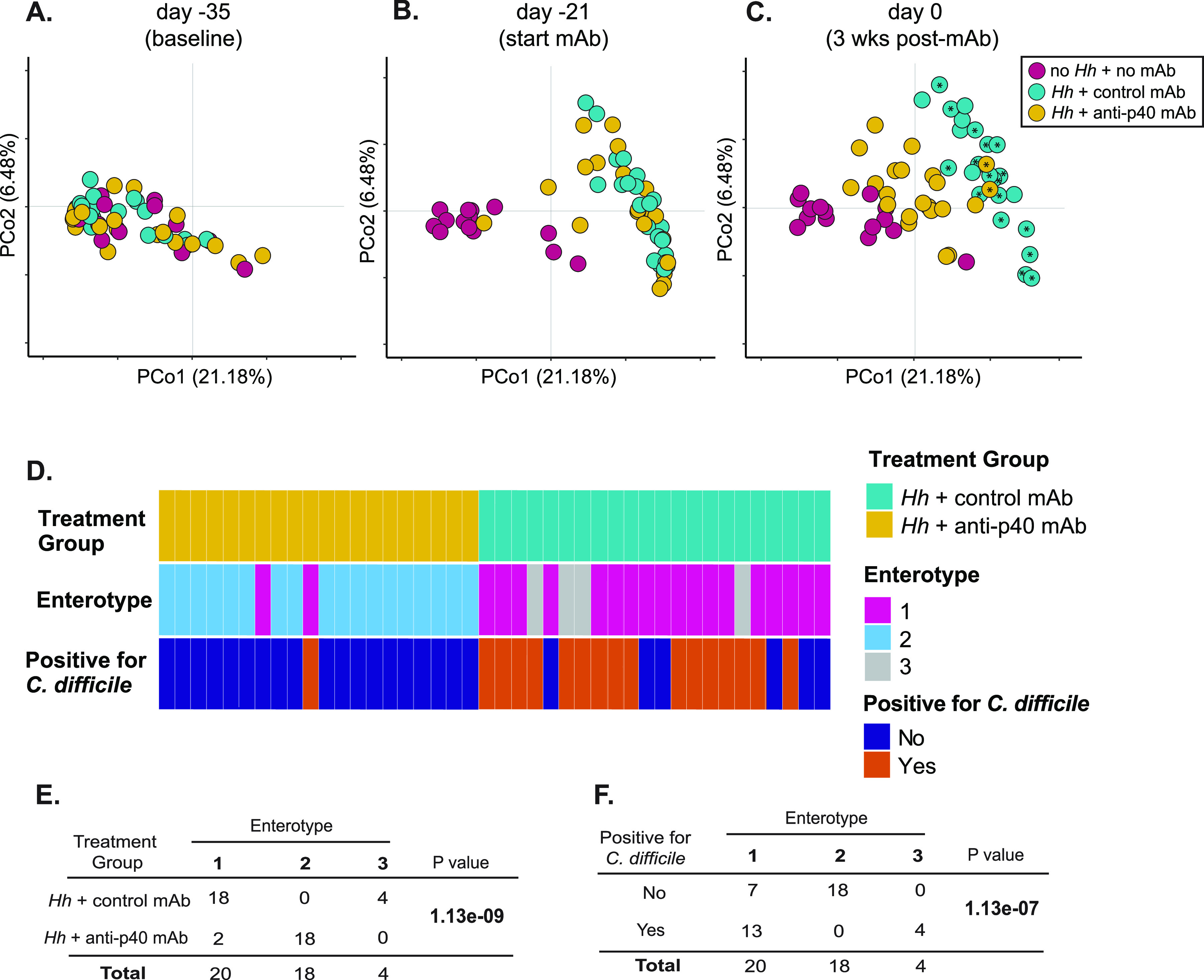
IBD-induced changes in microbiota structure are associated with susceptibility to C. difficile colonization. (A-C) Principal-component analysis plot of Theta YC distances of bacterial communities in feces collected from mice at (A) baseline (day -35), (B) 21 days prior to challenge with C. difficile (day -21; start of mAb treatment) and (C) day 0 of C. difficile challenge (after 3 weeks of mAb treatment). Data are from 3 independent experiments. *Hh* = H. hepaticus*; Cd* = C. difficile. Asterisked (*) symbols denote animals that went on to have detectable C. difficile in their intestinal contents at some point throughout the study. (D) Dirichlet multinomial mixtures (DMM) modeling of 16S rRNA sequences from feces collected from mice with IBD treated with control mAb or anti-p40 mAb on day 0 of C. difficile challenge. DMM allows for unbiased clustering of microbial communities into enterotypes, which can then be associated with outcomes of interest. Fecal samples from mice were partitioned into three enterotypes based on relative abundance of bacterial genera. The plot depicts treatment group, enterotype and C. difficile positivity 1-day post challenge for each animal, where each vertical segment of the plot refers to a single mouse. Samples are from 3 independent experiments. (E-F) Fisher’s exact tests on data presented in (C). Analyses revealed a significant association between enterotype and treatment group (E), as well as enterotype and whether animals became positive for C. difficile on day 1 post challenge (F). See also Fig. S5.

One day after spore challenge, 13/20 (65%) of mice that had received the isotype control mAb shed C. difficile in their feces (Fig. 1C, Fig. S2). By 9 days after spore challenge, 15/20 (75%) of these mice had shed C. difficile at some point throughout the experiment compared to 2/17 (11.8%) mice treated with anti-p40 mAb (Fig. 1C and D, Fig. S2). Thus, colitic mice treated with the control mAb were significantly more susceptible to C. difficile colonization than those treated with anti-p40 mAb (*P* = 0.0002 by a Fisher's exact test) (Fig. 2D). Following challenge with C. difficile, signs of clinical disease were mild in mice from both groups. Clinical disease scores did not differ between animals with IBD that had shed C. difficile during the experiment compared to animals that never shed C. difficile, regardless of which mAb they received (Fig. S3A). Similarly, histopathological analysis of cecal and colon tissue did not reveal a significant difference in the degree of edema, inflammatory infiltrate, and epithelial damage in C. difficile-susceptible and resistant mice within each treatment group (Fig. S3B–C), despite the reduction of inflammation in animals that received anti-p40 mAb. Collectively, these results confirm that inflammation creates a permissive landscape for C. difficile colonization and, when inflammation is decreased via anti-p40 mAb treatment, susceptibility to C. difficile is lost.

10.1128/mbio.01904-22.5 FIG S5 Abundance of top 10 bacterial taxa in feces of mice with gut microbial communities of enterotypes 1, 2, and 3. Log_10_ relative abundance of top 10 most abundant taxa in fecal samples collected from animals on the day of C. difficile challenge and colored by enterotype (Fig. 2D in main text). Box plots represent mean aggregated relative abundance of OTUs within each bacterial genus. Asterisks (*) denote unclassified genera. Dashed line represents the limit of detection, defined as the smallest relative abundance value in the dataset divided by 10. Data were analyzed via a Kruskal Wallis and Dunn’s test for each genus (*, *P* < 0.05; **, *P* < 0.01; ***, *P* < 0.001; ****, *P* < 0.0001). Download FIG S5, PDF file, 0.1 MB. Copyright © 2022 Barron et al. 2022 Barron et al. https://creativecommons.org/licenses/by/4.0/ This content is distributed under the terms of the Creative Commons Attribution 4.0 International license.

### IBD-induced changes in microbiota structure are associated with susceptibility to C. difficile colonization.

Given alterations in gut microbiota composition underlie the pathogenesis of both IBD and CDI (12, 29), we used 16S rRNA-encoding gene amplicon sequence analysis to examine the fecal microbiota of mice prior to H. hepaticus colonization (baseline), 21 days before challenge with C. difficile (when inflammation has developed and immediately prior to initiation of mAb treatment), and on the day of C. difficile challenge (after 3 weeks of mAb treatment). The microbiota of groups that were treated with mAb were compared to animals that were not colonized with H. hepaticus or treated with mAb. At baseline, all mice had a similar gut microbiota structure (Fig. 2A). The development of colitis was accompanied by alterations in microbial community composition (Fig. 2B). By the day of C. difficile challenge and following 3 weeks of mAb treatment, the microbiota of anti-p40 mAb-treated animals had largely shifted toward baseline (Fig. 2C). Animals that went on to become susceptible to C. difficile colonization clustered together, regardless of which mAb they received (see asterisked symbols in Fig. 2C). For example, two mice treated with anti-p40 mAb that became colonized by C. difficile (Fig. 1C) clustered with control mAb-treated animals that were also susceptible to the pathogen (Fig. 2C).

The differences in fecal microbiota structure between treatment groups were also observed in the cecal contents of animals harvested on the day of C. difficile challenge (Fig. S4A). Targeted analyses of cecal short-chain fatty acid (SCFA) concentrations revealed a significant decrease in butyrate concentrations, and increase in propionate levels, in animals with IBD treated with control mAb compared to mice without IBD (Fig. S4B). Though there was a trend toward increased butyrate concentrations in animals treated with anti-p40 mAb compared to those administered control mAb, this difference was not significant (Fig. S4B). Levels of cecal bile acids were similar between all treatment groups (Fig. S4C).

10.1128/mbio.01904-22.4 FIG S4 SCFA and bile acid concentrations in cecal contents of mice without IBD and mice with IBD treated with control or anti-p40 mAb. (A) Principal component analysis plot of Theta YC distances of bacterial communities in cecal contents collected from mice without colitis and with colitis after 3 weeks of receiving control mAb or anti-p40 mAb. (B) Concentrations of acetate, butyrate, and propionate in cecal contents of mice without IBD and with IBD treated with control mAb or anti-p40 mAb, as measured via LC-MS. Results for each compound were analyzed using an ANOVA followed by Tukey’s test (*, *P* < 0.05; **, *P* < 0.01). (C) LC-MS analysis of primary and secondary bile acids in cecal contents. Statistical significance was determined via an ANOVA followed by Tukey’s test or Kruskal Wallis with Dunn’s multiple comparison’s test depending on data distribution (*, *P* < 0.05). Asterisked (*) bile acids denote those only produced in mice. CA = Cholic acid; αMCA= Alpha muricholic acid; βMCA = Beta muricholic acid; GCA = Glychocholic acid; TCA=Taurocholic acid, UDCA = Ursodeoxycholic acid; DCA = Deoxycholic acid; HDCA= Hyodeoxycholic acid, *w*MCA = Omega muricholic acid. Download FIG S4, PDF file, 0.1 MB. Copyright © 2022 Barron et al. 2022 Barron et al. https://creativecommons.org/licenses/by/4.0/ This content is distributed under the terms of the Creative Commons Attribution 4.0 International license.

The observed changes in microbiota structure in colitic mice treated with anti-p40 mAb, and similarities in microbial community structure in animals susceptible to C. difficile colonization (Fig. 2C), prompted us to assess whether there is a relationship between microbiota composition and C. difficile susceptibility in the setting of IBD. To that end, we used Dirichlet multinomial mixtures (DMM) modeling to cluster the microbial communities in each animal into enterotypes based on the abundance of bacterial genera in their feces on the day of C. difficile challenge (Fig. 2D) (30). We grouped animals based on mAb treatment and subdivided them based on whether they had detectable levels of C. difficile in their feces 1 day after challenge with spores.

The DMM model with the highest likelihood (determined as having the lowest Laplace approximation value) partitioned samples into 3 enterotypes (Fig. 2D). Mice with communities in enterotype 1 were largely those with IBD treated with control mAb; animals with communities in enterotype 3 were solely mice in this group. Only mice treated with anti-p40 mAb had a microbiota of enterotype 2 (Fig. 2D). Examination of the top 10 most abundant genera within samples revealed that, compared to enterotypes 1 and 3, enterotype 2 communities exhibited significantly higher concentrations of *Lachnospiraceae*, *Ruminoccoacceae*, and *Alistipes* (Fig. S5). Both enterotype 1 and 3 microbiota were characterized by increased abundances of *Enterobacteriaceae*, *Lactobacillus*, and *Bifidobacterium* relative to enterotype 2. Additionally, compared to the other two enterotypes, the relative abundance of *Erysipelotrichaceae* and *Akkermansia* were increased in enterotype 1 communities (Fig. S5). Interestingly, enterotypes were significantly correlated with treatment groups and C. difficile susceptibility (Fig. 2E and F). Accordingly, all animals that carried an enterotype 3 microbiota, and a majority that harbored communities of enterotype 1, were susceptible to C. difficile on day 1 post challenge (Fig. 2D). All mice with enterotype 2 communities were resistant to C. difficile colonization (Fig. 2D). These results indicate there is an association between microbiota structure and C. difficile susceptibility in the setting of intestinal inflammation.

### The microbiota from animals with active colitis is sufficient to transfer susceptibility to C. difficile.

**FIG 3:**
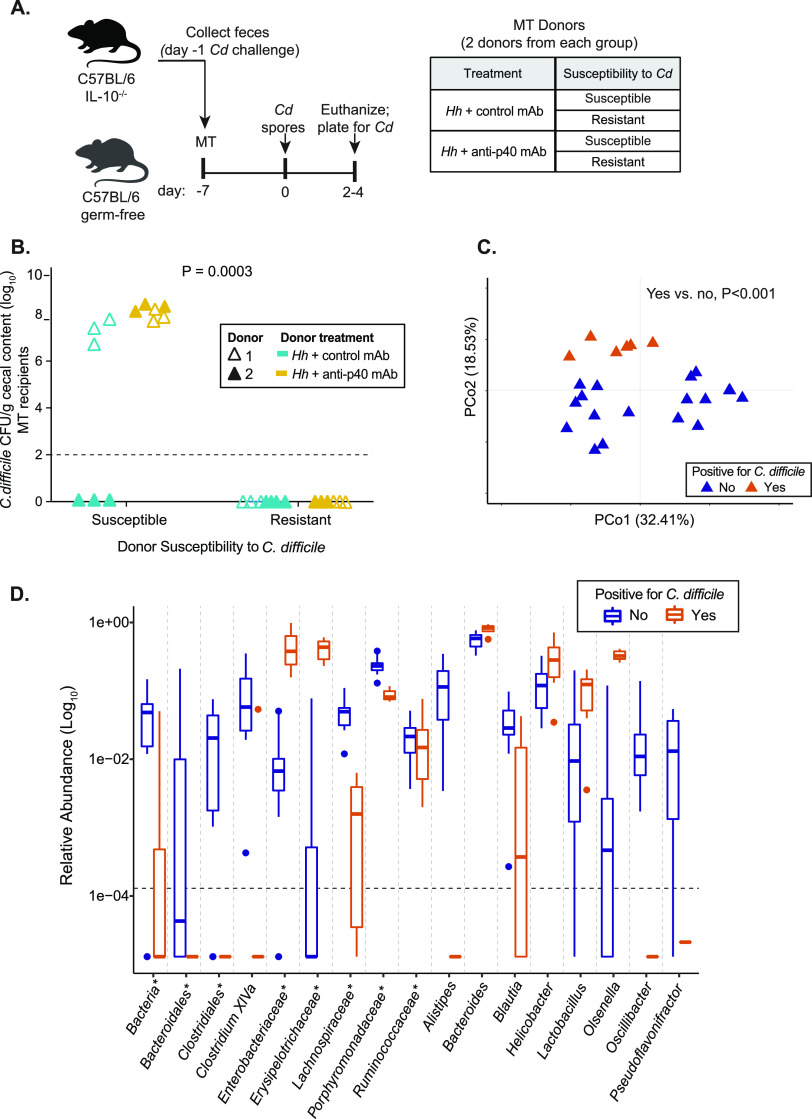
The microbiota from animals with active colitis is sufficient to transfer susceptibility to C. difficile. (A) Experimental design. Wild-type C57BL/6 germfree mice were administered feces collected from colitic IL-10^−/−^ mice treated with control mAb or anti-p40 mAb and suspended in PBS via oral gavage. Donor feces were collected 1 day prior to C. difficile challenge; donors included animals that went on to become susceptible or remain resistant to the pathogen. Two independent experiments were completed with different donors each time (i.e., donor 1 and donor 2). 1-week post microbiota transfer (MT), recipients were challenged with C. difficile spores. Animals were euthanized on days 2 to 4 post challenge. (B) C. difficile concentrations in the cecal contents of gnotobiotic mice at the time of sacrifice (days 2 to 4 post challenge). Data are from 2 independent experiments. A Fisher’s exact test demonstrated a significant association between MT donor and recipient susceptibility to C. difficile colonization. (C) Principal-component analysis plot of Theta YC distances of bacterial communities in feces collected from MT recipients on day 0 of C. difficile challenge (7 days post transfer). C. difficile positivity indicates whether animals went on to become colonized by, or remain resistant to, the pathogen by the end of the experiment. An AMOVA revealed a significant difference in microbiota structure between susceptible and resistant mice. (D) Differentially abundant bacterial taxa in the feces of MT recipient mice on day 0 C. difficile challenge. Linear discriminant analysis effect size analysis (LEfSe) analysis was used to identify OTUs that were differentially abundant between recipients that were susceptible or resistant to C. difficile. Box plots represent mean aggregated log_10_ relative abundance of OTUs with an LDA score of ≥2 for each bacterial genus. Asterisks (*) denote unclassified genera. The dashed line represents the limit of detection, defined as the smallest relative abundance value in the data set divided by 10. See also Fig. S6.

Our data indicate that alterations in the composition of the indigenous gut microbiota are associated with increased susceptibility to C. difficile. These changes largely correspond with treatment status, and thus the degree of inflammation at the time of challenge with C. difficile spores. To separate the development of inflammation and the associated changes in the community structure of the microbiota, we determined if the specific microbiota composition seen in colitic animals conferred susceptibility to C. difficile in the absence of inflammation. To do this, we conducted microbiota transfer (MT) experiments in C57BL/6 germfree mice. Donor animals included colitic mice treated with control mAb or anti-p40 mAb. We selected donors from each treatment group that were either susceptible or resistant to C. difficile (Fig. 3A) to capture potential variability or features in microbiota structure that may underlie resistant and susceptible phenotypes, regardless of which treatment the animal that harbored the microbiota had received.

10.1128/mbio.01904-22.6 FIG S6 Fecal lipocalin-2 concentrations, survival, and clinical scores of microbiota transfer (MT) recipients. (A) Fecal lipocalin-2 levels of MT recipient mice at baseline and 7 days post transplant. Mice exhibited lower lipocalin-2 concentrations relative to IL-10^−/−^ mice with IBD (Fig. 1B in main text). (B) Survival curve of MT recipients. Several animals harboring microbiota from C. difficile susceptible donors died prior to the experiment endpoint. (C) Clinical disease of MT recipients at the time of sacrifice. Animals colonized by C. difficile exhibited overt signs of disease. *Hh =* H. hepaticus*, Cd =* C. difficile. Download FIG S6, PDF file, 0.2 MB. Copyright © 2022 Barron et al. 2022 Barron et al. https://creativecommons.org/licenses/by/4.0/ This content is distributed under the terms of the Creative Commons Attribution 4.0 International license.

Feces were collected from donor animals 1 day prior to C. difficile spore challenge (Fig. 3A) and stored until the results of challenge were known (Fig. 2D). Subsequently, the fecal pellets were thawed, suspended in PBS, and administered to recipient germfree animals via oral gavage (Fig. 3A). The donor microbiota was allowed to engraft in recipient animals for 7 days (Fig. 3A). We used wild-type animals (IL-10^+/+^) as recipients because they do not develop colitis when colonized with H. hepaticus (10). This was confirmed by the low levels of lipocalin-2 detected in the stool of the MT recipients 1-week post transfer (Fig. S6A), compared to H. hepaticus-colonized IL-10^−/−^ SPF mice (Fig. 1B). One week post-MT, animals were challenged with C. difficile spores and colonization status was determined on days 2 to 4 post infection (Fig. 3A), depending on the onset of severe clinical disease (Fig. S6B).

Interestingly, recipient mice receiving MT from three of the four susceptible donors also exhibited high C. difficile burdens in their intestinal contents (Fig. 3B). A Fisher’s exact test demonstrated a significant association between MT donor and recipient susceptibility to C. difficile colonization (*P* = 0.0003). Notably, animals positive for C. difficile exhibited overt disease, and the clinical scores were higher on average than those observed in IL-10^−/−^ mice with IBD and susceptible to colonization by C. difficile (Fig. S3A and Fig. S6C), perhaps pointing to a protective immune phenotype in animals with preexisting intestinal inflammation. Animals that received microbiota from all 4 resistant donors were subsequently resistant to C. difficile colonization (Fig. 3B).

Examination of the microbiota on the day of C. difficile challenge revealed a clear difference in community composition between susceptible and resistant MT recipients, regardless of donor susceptibility (Fig. 3C). Linear discriminant effect size (LEfSe) analysis (31) was used to identify bacterial taxa that differed significantly between MT recipients that were susceptible to C. difficile colonization and those that were resistant. Taxa enriched in susceptible animals included members of the *Lactobacillus*, *Enterobacteriaceae*, *Olsenella*, *Helicobacter*, *and Erysipelotrichaceae* genera; resistant animals had higher abundances of *Lachnospiraceae*, *Porphyromonodaceae*, *Clostridiales*, *Clostridium XIVa*, *Ruminoccocaceae*, *Alistipes*, and *Blautia*, among others (Fig. 3D). Together, these data demonstrate that the microbiota associated with IBD drives susceptibility to C. difficile colonization in the absence of active intestinal inflammation.

### Machine learning models predict susceptibility to C. difficile based on microbiota composition.

Our results consistently demonstrate the critical role of the gut microbiota in regulating C. difficile colonization in the context of preexisting intestinal inflammation. Across experiments, we observed several taxa whose abundances consistently differed between animals susceptible or resistant to C. difficile colonization (e.g., *Enterobacteriaceae* and *Lachnospiraceae*, respectively). Based on these observations, we sought to determine whether there were global features of the microbiota associated with susceptibility and resistance to C. difficile. To that end, we developed random forest models to predict animals’ susceptibility to C. difficile colonization based on fecal microbiota composition on the day of spore challenge.

**FIG 4:**
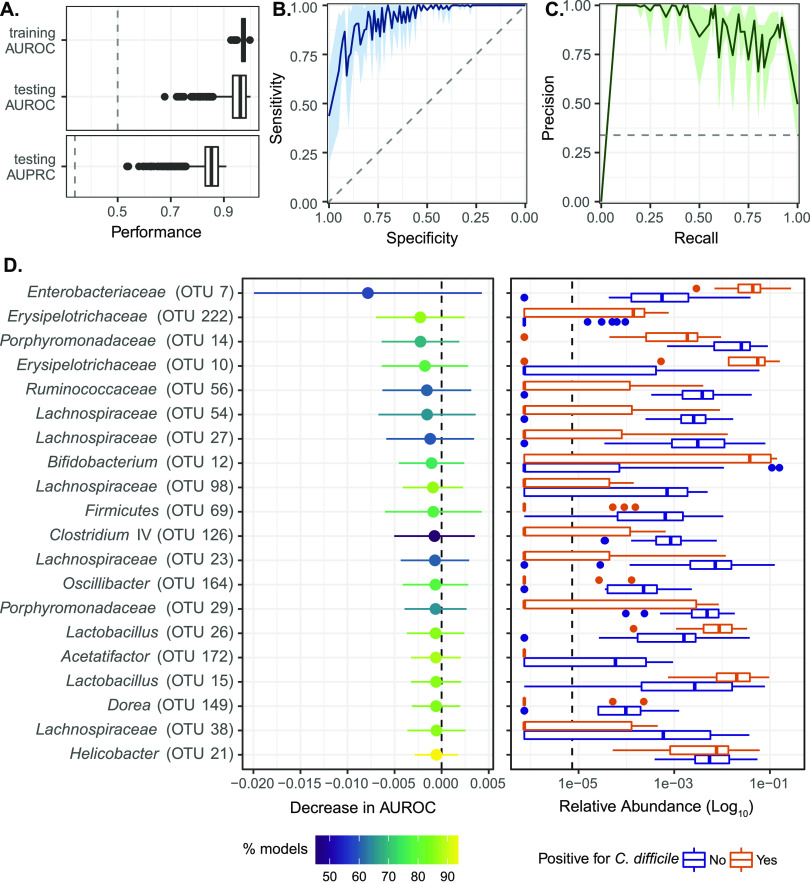
Machine learning models predict susceptibility to C. difficile based on microbiota composition. (A) Mean area under the receiver-operator characteristic curve (AUROC) on the cross-validation folds during model training, mean AUROC on the held-out test data, and mean area under the precision-recall curve (AUPRC) on the held-out test data. The dashed lines represent the baseline AUROC (0.5) and AUPRC (0.34). (B) Receiver-operator characteristic curve for the test data, with mean specificity plotted against sensitivity. The light green shaded area shows the standard deviation. (C) Precision-recall curve for the test data, with mean precision plotted against recall. The light blue shaded area shows the standard deviation. (D) Top 20 most important OTUs as determined by permutation feature importance (left panel). OTUs with a greater decrease in AUROC when permuted are more important. The points represent the median decrease in AUROC with the tails as the standard deviation. Color represents the percentage of models for which an OTU’s permutation AUROC was significantly different from the actual AUROC (*P* < 0.05). The right panel depicts the log_10_-transformed relative abundance for the top 20 most important OTUs on day 0 of the experiment, colored by C. difficile presence on day 1. The dashed line represents the limit of detection, defined as the smallest relative abundance value in the data set divided by 10. See also Table S2.

Our analysis combined samples from five independent experiments, three of which were conducted in IL-10^−/−^ mice treated with control mAb or anti-p40 mAb and challenged with C. difficile (Fig. 1 and 2). The remaining two experiments involved germfree MT mice, as outlined above (Fig. 3). In total, model development utilized 62 animals, 21 of which were susceptible to C. difficile colonization (34%) and 41 that were resistant (66%). To account for variation in experiment endpoints, we developed models that could classify mice as having detectable C. difficile in their feces 1 day post challenge. Communities characterized by 16S rRNA amplicon sequence analysis were grouped by experiment and randomly partitioned into training and test data sets (65% and 35% of samples, respectively). Model performance was evaluated by measuring the area under the receiver operator characteristic curve (AUROC) (Fig. 4A and B), as well as area under the precision recall curve (AUPRC) for the test data (Fig. 4A and C). Both metrics supported high predictive performance (Fig. 4A to C).

10.1128/mbio.01904-22.8 TABLE S2 Feature Importance analyses, top 20 OTUs (corresponds with Fig. 4D in main text). OTUs are ranked by mean decrease in AUROC after permutation. Download Table S2, PDF file, 0.03 MB. Copyright © 2022 Barron et al. 2022 Barron et al. https://creativecommons.org/licenses/by/4.0/ This content is distributed under the terms of the Creative Commons Attribution 4.0 International license.

Given the good predictive value of the models, we next sought to identify operational taxonomic units (OTUs) that were most important in predicting C. difficile susceptibility using permutation importance (32, 33). The top 20 OTUs are depicted in Fig. 4D. OTU 7, a member of the *Enterobacteriaceae* genus, had the strongest effect on AUROC in this permutation analysis and was significant (*P* < 0.05) in 58% of the trained models (Fig. 4D, Table S2). OTUs that significantly decreased performance for >80% of all trained models included those belonging to the *Erysipelotrichacea*e, *Lachnospiraceae*, *Lactobacillus*, *Acetatifactor*, *Dorea*, and *Helicobacter* genera (Fig. 4D, Table S2). Several OTUs were significant across ~60 to 80% of the trained models, including those within the *Porphyromonodaceae*, *Lachnospiraceae*, *Ruminococcaeae*, and *Bifidobacterium* genera (Fig. 4D, Table S2).

Plotting the relative abundance of these top 20 OTUs in fecal samples collected from animals on the day of C. difficile challenge revealed clear differences between those that became positive, or remained negative, for the pathogen 1 day post challenge (Fig. 4D). For example, all OTUs belonging to the *Lachnospiraceae* and *Porphyromonodaceae* genera were enriched in resistant animals whereas *Enterobacteriaceae*, *Lactobacillus*, and *Erysipelotrichaceae* were more abundant in susceptible mice. These findings highlight specific microbiota taxa that are associated with modulating susceptibility to C. difficile colonization.

## DISCUSSION

The indigenous gut microbiota plays a central role in colonization resistance to C. difficile, in part through interactions with the host (8–11, 29). Murine models have been critical for advancing our understanding of the structural and functional aspects of the microbiota that regulate susceptibility to CDI (34). However, most murine studies have focused on C. difficile colonization in the context of antibiotic treatment (4, 24). There is a need to develop appropriate models to understand mechanisms underlying nonantibiotic risk factors for CDI development, including underlying IBD.

Here, we demonstrate that intestinal inflammation alters microbial community structure in mice with colitis, and that these changes are associated with a loss of colonization resistance to C. difficile. We show that inflammation sculpts the microbiota to permit C. difficile colonization, though it is not required in addition to, or instead of, these alterations. These findings suggest a temporal relationship between the development of inflammation and loss of microbiota-mediated resistance to C. difficile. Collectively, our results provide a new perspective on the relative contributions of the host and microbiota in colonization resistance to C. difficile. Importantly, they support the primacy of the microbiota in regulating C. difficile susceptibility, as described for antibiotic-associated CDI pathogenesis.

We provide a novel system to study features of the microbial community modulating vulnerability to CDI in the setting of intestinal inflammation. Notably, many of the taxa we consistently identified as associating with susceptibility to C. difficile colonization have previously been implicated in CDI. For example, in agreement with our studies, higher concentrations of *Lactobacillaceae*, *Erysipelotrichaceae*, and *Enterobacteriaceae* are generally associated with susceptibility to CDI in murine and human systems (3, 35–37). In contrast, *Lachnospiraceae*, *Ruminococcaceae*, and *Porphyromonodaceae*, which were enriched in resistant animals, are linked with protection against, or clearance of, C. difficile in mouse models of CDI (3, 37–41). Regarding infection in humans, these taxa are more abundant in healthy people compared to patients with CDI (36). Our findings, in conjunction with these studies, suggest there are microbial signatures that correlate with susceptibility to CDI in various intestinal contexts. Nevertheless, while a single taxon may be specifically associated with susceptibility or resistance to C. difficile within a given microbial community, if and how it modulates susceptibly in the setting of *any* community is unclear.

For instance, several taxa associated with resistance to CDI in our study and others (e.g., *Lachnospiraceae* and *Ruminococcaceae*) shape the intestinal metabolic milieu in ways that could interfere with C. difficile colonization, such as through the production of SCFAs and secondary bile acids (3, 42–47). Previous work has demonstrated that antibiotics alter intestinal concentrations of these compounds (46, 48, 49) and these alterations, particularly in bile acid metabolism, support C. difficile growth (46). Interestingly, we found no significant differences in cecal SCFA and bile acid profiles between colitic mice treated with control mAb and anti-p40 mAb, despite variations in microbiota structure and C. difficile susceptibility observed between these groups. These data suggest that, in this system, susceptibility to CDI is likely influenced by factors other than, or in addition to, these compounds (50). Thus, it is becoming clear that the relationship between C. difficile the intestinal environment, as it is shaped by both the microbiota and the host, is a complex, multilayered system of multiple interactions (51). Understanding the functional significance of microbiota members in CDI susceptibility will require integrative experimental frameworks that account for this dynamic network of interactions (51).

From a clinical standpoint, our findings are intriguing considering the intersection between IBD and risk for CDI (22). People with IBD have altered gut microbial community structure compared to healthy individuals (13). Accordingly, many of the differences in microbiota composition observed in patients with IBD, such as loss of *Lachnospiraceae* and enrichment in *Enterobacteriaceae* species, correspond with those described here, and by others, as being linked with CDI development (52, 53). Thus, our study suggests the IBD-associated inflammatory landscape promotes formation of a susceptible microbiota structure while hindering expansion of resistance-associated bacteria. Therefore, controlling IBD-associated inflammation may alter the microbiota in ways that restrict C. difficile growth. Further explorations of the host-microbiota-CDI interface in the setting of IBD in murine models and patients will reveal context-dependent mechanisms of C. difficile pathogenesis most relevant to patients with IBD. These studies are critical in the development of prophylactic and therapeutic strategies for managing CDI in IBD patients.

## MATERIALS AND METHODS

### Bacterial culture.

Helicobacter hepaticus strain 3B1 (ATCC 51448) was streaked onto tryptic soy agar (TSA; Hardy Diagnostics) blood plates containing 5% sheep’s blood and grown for 3 to 4 days in a vinyl hypoxic chamber (2% O_2_, 5% CO_2;_ Coy Industries) maintained at 37°C. For murine inoculations, H. hepaticus from actively growing plates was streaked onto fresh blood agar and grown an additional 3 to 4 days. Each plate was washed with sterile tryptic soy broth (TSB; Spectrum Chemical) and bacterial suspensions were combined and centrifuged at 4,000 × *g* for 10 min. The resulting pellet was resuspended in fresh TSB for oral gavage of mice.

To prepare C. difficile spore stocks, C. difficile VPI10463 was grown overnight at 37°C in 5 mL of Columbia Broth in a vinyl anaerobic chamber (Coy Industries). Three overnight cultures were prepared. The following day, the cultures were each added to 40 mL of Clospore media (54) and incubated at 37°C for 14 days. After this time, the tubes were centrifuged at 4°C and washed 2× in ice-cold sterile water. After another wash in cold 0.05% Tween 20, the pellets were combined and washed again. The pellet was suspended in 1 mL of cold 50% Histodenz gradient (Sigma-Aldrich) diluted in distilled, nuclease-free water and carefully pipetted into a conical tube containing an additional 19 mL of Histodenz. After centrifugation for 20 min, the spores were washed with 0.05% Tween 20 once, then ice-cold water 3 more times. The final pellet was suspended in water and transferred to a microcentrifuge tube for storage at 4°C. Spore purity was verified via phase-contrast microscopy. Spore inocula for mouse infections were prepared as previously described (24). The inocula were quantified via serial plating on prereduced cycloserine-cefoxitin-fructose agar containing 0.1% taurocholate (TCCFA), both at the time of preparation (3 days before challenging mice) and after infection. TCCFA was prepared as previously described (55).

### Mice.

All animal experiments were completed with approval from the University of Michigan Committee on Use and Care of Animals. Male and female C57BL/6 IL-10-deficient mice, aged 6 to 11 weeks at the start of experiments, were used. Cohoused littermates of same sex were randomly assigned to experimental groups. All mice were from a breeding colony obtained from Jackson Laboratories over 20 years ago. Animals were housed in specific-pathogen-free (SPF), *Helicobacter*-free conditions at 22°C under a 12h light/dark cycle, and animal husbandry was performed in an AAALAC-accredited facility. For microbiota transfer experiments, male and female germfree C57BL/6Ncr mice aged 7 to 11 weeks were obtained from a colony established and maintained by the University of Michigan Germ-Free Mouse Facility. Cohoused littermates of the same sex were randomly assigned to experimental groups and mice received sterile food, water, and bedding throughout the experiments.

### Helicobacter hepaticus colitis.

To trigger colitis in IL-10^−/−^ mice, animals were inoculated with ~10^8^ CFU (CFU) of H. hepaticus in 100 μL volume TSB via oral gavage (56). Control mice received sterile TSB. H. hepaticus colonization was confirmed by PCR with primers specific for the H. hepaticus 16S rRNA gene (57) (Table S1) using DNA isolated from feces 5 to 7 days post inoculation. For extractions, feces were homogenized in 500 μL sterile PBS (Gibco) and the homogenate centrifuged at 500 × *g* for 10 to 15 secs. DNA was extracted from the resulting supernatant using DNeasy UltraClean Microbial kit (Qiagen), following manufacturer’s instructions.

10.1128/mbio.01904-22.7 TABLE S1 Primers and probes used for PCR and RT-PCR analyses. Download Table S1, PDF file, 0.1 MB. Copyright © 2022 Barron et al. 2022 Barron et al. https://creativecommons.org/licenses/by/4.0/ This content is distributed under the terms of the Creative Commons Attribution 4.0 International license.

### Monoclonal antibody treatments.

Anti-p40 (Bio X Cell catalog number BE0051, RRID: AB_1107698) and mouse IgG2A isotype control (Bio X Cell catalog number BE0089, RRID: AB_1107769) monoclonal antibodies were diluted in *InVivo*Pure pH 7.0 Dilution Buffer (Bio X Cell). Mice were injected intraperitoneally with 1 mg of antibody suspended in 200 μL buffer every 3 to 4 days for 3 weeks, beginning 14 days post H. hepaticus inoculation. The frequency of injections was determined based on preliminary studies. Mice without colitis received 200 μL of dilution buffer at each time point. Mice challenged with C. difficile continued to receive antibodies after infection until the end of experiment.

### C. difficile infections and quantification from intestinal content.

Animals were administered ~3 × 10^4^ C. difficile strain VPI10463 spores in 40 to 50 μL water or mock-challenged with water by oral gavage. Mice were monitored for signs of clinical disease. Disease scores were averaged based on scoring of the following features for signs of disease: weight loss, activity, posture, coat, diarrhea, and eyes/nose. A 4-point scale was assigned to score each feature, and the sum of these scores determined the clinical disease severity score (58).

To measure C. difficile colonization, fecal pellets were collected in preweighed sterile tubes. After collection, tubes were reweighed to determine fecal weight, and passed into a vinyl anaerobic chamber (Coy Industries). Feces were diluted 1:10 (wt/vol) in prereduced sterile PBS (Gibco). The fecal homogenate was serially diluted in PBS and 100 μL was plated on prereduced TCCFA; the same procedure was followed for plating cecal contents at necropsy. TCCFA plates with fecal or cecal samples were incubated at 37°C for at least 18 h prior to colony enumeration.

### Clinical disease severity and histopathology.

At the end of experiments, mice were euthanized via CO_2_ asphyxiation and colon and cecal tissues were collected and fixed in 10% formalin and stored in 70% ethanol until processing. Tissue was embedded in paraffin and sectioned to generate hematoxylin and eosin-stained slides. To assess colitis severity resulting from H. hepaticus colonization, slides were scored for lymphocytic inflammation by a board-certified veterinary histopathologist (I.L.B.) blinded to the experimental groups using a 4-point scoring system, as previously described (59). Histopathologic damage associated with C. difficile infection was scored using epithelial destruction, immune cell infiltration, and edema on 4-point scale and the sum of all categories was used to determine histological score (24, 35).

### Microbiota transfer experiments.

Inocula for microbiota transfers (MT) were prepared as follows. Fecal pellets were collected in preweighed tubes from donor mice 1 day prior to challenge with C. difficile and frozen at −80°C. Donors included colitic IL-10^−/−^ mice treated with control mAb or anti-p40 mAb that went on to become susceptible or remain resistant to C. difficile colonization. On the day of MT, donor fecal pellets were thawed, reweighed, and passed into an anaerobic chamber. Feces were diluted 1:20 (wt/vol) in prereduced PBS (Gibco) and vortexed for 10 min at maximum speed. Samples were centrifuged at 800 rpm for 30 secs. 50 to 70 μL of the supernatant was administered to germfree animals via oral gavage. After inoculation, the gavage material was plated on blood agar plates and incubated overnight in an anaerobic chamber (37°C) to confirm the presence of viable bacteria. After 7 days, mice were challenged with C. difficile and colonization was monitored as described above.

### Fecal lipocalin-2 quantification.

Fresh fecal pellets were collected from mice at baseline, 2 weeks post H. hepaticus inoculation, and after 3 weeks of monoclonal antibody treatment in preweighed microcentrifuge tubes. Tubes were reweighed to determine fecal weight and the pellet suspended in PBS (Gibco) with 0.1% Tween 20. Suspensions were vortexed at maximum speed for 20 min to homogenize the sample and centrifuged at 4°C for 10 min. The supernatant was collected and used to quantify fecal lipocalin-2 levels using the mouse lipocalin-2/NGAL DuoSet ELISA kit (R&D Systems) in a microplate reader according to manufacturer’s instructions.

### Tissue RNA extraction and RT-PCR.

Colon tissue snips collected at time of necropsy were placed in RNA*later* solution (Invitrogen) and stored overnight at 4°C, then transferred to −80°C until processing. Tissues were weighed, homogenized, and total RNA was extracted from samples using the AllPrep DNA/RNA minikit (Qiagen) according to manufacturer’s instructions. After extraction, RNA was diluted to 50 ng/μL for RT-PCR reactions.

For one-step RT-PCR analyses, 25 μL reaction mixtures were prepared using the TaqMan RNA-to-Ct™ 1-Step kit (Thermo Fisher) per manufacturer instructions, with 150 ng of RNA used per reaction. All reactions were run in technical duplicates with appropriate controls. RT-PCR was performed on a LightCycler96 qPCR machine (Roche) with initial incubations at 48°C for 15 min and 95°C for 10 min, followed by 40 cycles of 95°C for 15s and 60°C for 1 min. Relative expression of targets was determined via the 2 ^ΔΔ^*^CT^* method, using β-actin as the control gene. Primers used for 1-step RT-PCR are depicted in Table S1. All probes were modified with FAM and TAMRA at the 5′ and 3′ ends, respectively.

### Targeted metabolomics.

Quantification of cecal short-chain fatty acids (SCFA) and bile acids was completed by the University of Michigan Medical School Metabolomics Core. Cecal contents were collected in preweighed sterile tubes, immediately frozen in liquid nitrogen, and stored at −80°C until submission. Prior to submission, tubes were reweighed to determine weight of cecal contents, and approximately 50 mg submitted for use in subsequent assays.

**(i) SCFAs.** Water and acetonitrile containing internal standards (500 μM D4-acetic acid, 250 μM D7-butyric acid, and 6.25 μM D11-hexanoic acid) were added to each sample. Samples were homogenized (Branson) and centrifuged at 15,000 × *g* at 4°C for 10 min. Supernatant was transferred to a 1.8 mL glass autosampler vial and 200 mM 3-nitrophenylhydrazine (3-NPH) in 1:1 acetonitrile: water and 120 mM 1-Ethyl-3-(3-dimethylaminopropyl) carbodiimide in 1:1 acetonitrile water with 6% pyridine were added. Samples were vortexed and placed in a warming oven at 40°C for 30 min.

Once derivatization was complete, samples were cooled, and 90/10 water/acetonitrile was added. Standards were prepared identically to cecal samples, substituting volatile fatty acid mix (Sigma-Aldrich) diluted to concentrations ranging from 3 μM to 3000 μM. Samples were analyzed via liquid chromatography mass spectrometry (LC-MS) using an Agilent 1290 LC coupled to an Agilent 6490 triple quadrupole MS and a Waters HSS T3, 2.1 mm x 100 mm, 1.8 μm particle size chromatographic column. Mobile phase A contained 0.1% formic acid in water while mobile phase B consisted of 0.1% formic acid in methanol. The gradient was as follows: linear ramp from 15% to 80% B from 0 to 12 min; step to 100% B from 12 to 12.1 min; hold 100%B from 12.1 to 16 min; step to 15% B from 16 to 16.1 min; hold 15% B from16.1 to 20min. The injection volume was 5 μL and the column temperature was 55°C. MS parameters were as follows: gas temperature 325°C, gas flow 10 L/min, nebulizer 40 lb/in^2^, capillary voltage 4000V, scan type MRM, negative ion mode, delta EMV 600. Quantitation was performed using MassHunter Quantitative Analysis software (Agilent v.B.07.00) by measuring the ratio of peak area of the 3-NPH derivatized SCFA species to its closest internal standard (by retention time). Linear standard curves were used to estimate SCFA concentrations in the extract, which were normalized to the measured mass of cecal contents.

**(ii) Bile acids.** To determine bile acid concentrations, extraction solvent containing chilled acetonitrile with 5% NH_4_OH and isotope-labeled internal standards were added to 20 mg of cecal samples. Samples were homogenized by probe sonication for 20 secs. The homogenized mixture was centrifuged, and an aliquot of supernatant transferred to an LC-MS autosampler and dried in a speedvac set to 45°C for approximately 45 min. Samples were reconstituted in 50/50 methanol/water. A series of calibration standards ranging from 0 to 1000 nM were prepared along with samples for metabolite quantification.

Bile acid LC-MS analyses were performed on an Agilent 1290 LC coupled with a 6490 Triple Quad mass spectrometer operated in MRM mode. Metabolites were separated on a HSS T3, 2.1 mm x 50 mm, 1.8m particle size column using water + 0.1% formic acid as mobile phase A, and acetonitrile + 0.1% Formic acid, as mobile phase B. The flow rate was 0.35 mL/min with the following gradient: linear from 5% to 25% B over 2 min, linear from 25% to 35% B over 16 min, linear from 35% to 75% B over 8 min, followed by isocratic elution at 95% B for 8 min. The system was returned to starting conditions (5% B) in 0.1 min and held there for 3 min to allow for column reequilibration before injecting another sample. Data were processed using MassHunter Quantitative analysis. Metabolites were quantitated by determining the ratio of the peak area for each compound to that of the closest-matching internal standard (by RT), and then calculating concentration using a 5-point linear calibration curve prepared using authentic standards. Calibration curve accuracy was determined to be better than 80% for each standard. Measured bile acid concentrations were normalized to dry sample weight after quantification.

### 16S rRNA-encoding gene amplicon sequencing and analysis.

The University of Michigan Microbiome Core extracted total DNA from intestinal contents, including either whole fecal pellets or 200 to 300 μL of contents diluted 1:10 (wt/vol) in sterile PBS using the MagAttract PowerMicrobiome kit (Qiagen), and prepped DNA libraries as previously described (60). Samples were randomized into each extraction plate. DNA was amplified using dual-index primers targeting the V4 region of the 16S rRNA gene, as described previously (61). Sequencing was conducted on the Illumina MiSeq platform using the MiSeq reagent kit V3 for a total of 500 total cycles, with modifications found in the Schloss SOP (https://github.com/SchlossLab/MiSeq_WetLab_SOP).

To assess sequencing error, the V4 region of the ZymoBIOMICS Microbial Community DNA standard (Zymo Research) was also sequenced. Data were analyzed using mothur (v.1.44.2) software package (62). Briefly, following assembly, filtering, and trimming, contigs were aligned to the Silva v.128 16S rRNA database. Any sequences that failed to align, or were flagged as possible chimeras by UCHIME, were removed (63). Sequences were clustered into operational taxonomic units (OTUs) with Opticlust (64) using a 97% similarity cutoff and classified via a Bayesian classifier using the Silva rRNA database. LEfSe analysis was conducted in mothur using the “lefse” command with default settings. The limit of detection for relative abundance analyses was calculated as the smallest nonzero relative abundance value observed in the data set divided by 10.

### Computational modeling and machine learning analyses.

Dirichlet multinomial mixtures (DMM) modeling (30) was used to achieve an unbiased analysis of the association between the microbiota at the time C. difficile first contacts the gut environment, and downstream susceptibility or resistance to the pathogen in mice with IBD treated with control mAb or anti-p40. Analyses included samples from 3 independent experiments, 2 in which mice were harvested day 9 post C. difficile challenge, and 1 where animals were sacrificed 1 day post challenge. Animals were classified based on whether they were positive for C. difficile 1 day post challenge to include samples from all experiments. Additionally, temporal colonization experiments revealed that positivity for C. difficile 1 day post challenge was associated with a long-term colonization phenotype, suggesting that this was a strong readout of robust colonization. DMM was completed in mothur using the “get.communitytype” command with default settings.

Supervised machine learning was performed according to the best practices outlined by Topçuoğlu et al., 2020 and implemented in the mikropml R package v1.2.1 (65). Models were trained on relative abundance data from fecal samples collected from animals on the day of C. difficile challenge to predict the presence of the pathogen on day 1 post challenge. The data were first preprocessed by centering and scaling abundance counts, collapsing perfectly correlated OTUs, and removing OTUs with 0 variance. For 100 random seeds, the data were randomly split into training and testing sets with 65% and 35% of the samples in each, respectively. Random forest models were trained on the training sets using 5-fold cross-validation to select the best hyper-parameter value (mtry: the number of OTUs included per tree), then the best models were evaluated on the held-out test sets by computing the AUROC and AUPRC. AUPRC is a useful metric for evaluating binary classifiers when there is an imbalance in the number of positive and negative events in a data set (i.e., a larger fraction of animals negative for C. difficile day 1 post challenge compared to positive) (66). An AUROC of 1 indicates the model perfectly distinguishes between sample groups, while an AUROC of 0.5 indicates the model does not predict better than random chance. For AUPRC, the baseline performance is calculated as the number of positive samples over the total number of samples, or, in this case, 0.34.

The most important OTUs contributing to model performance were determined by permutation feature importance tests (32). First, perfectly correlated OTUs were collapsed into a single representative. Then for each trained model, each OTU in the test data set was randomly shuffled 100 times and the new permutation performance (AUROC) was measured. A given OTU was considered significantly important for a model at an alpha level of 0.05, where less than 5% of the permutation AUROC values were greater than the original test AUROC. The OTUs that decreased the AUROC the most when permuted were considered the most important for model performance.

### Statistical analyses.

Statistical analyses were performed in Prism (GraphPad Software) or R. For comparing nonnormally distributed data a Kruskal Wallis test followed by Dunn’s multiple-comparison test, or Mann-Whitney U test, were performed. Data with normal distribution were analyzed using an ANOVA coupled with a Tukey’s *post hoc* test. We employed a Fisher's exact test to determine the relationship between C. difficile colonization susceptibility and IBD treatment group. A Fisher’s exact test revealed correlations between microbiota enterotype, treatment group, and C. difficile positivity. Differences in Theta YC distances between MT recipients that were susceptible or resistant to C. difficile were analyzed using analysis of molecular variance (AMOVA). For all analyses, a *P* value of less than 0.05 was considered statistically significant. Adobe Illustrator CC 2020 was used to arrange panels, modify color schemes as needed, and generate final figures.

### Data availability.

The workflow used to perform the machine learning analysis is available at https://github.com/SchlossLab/Barron_IBD-CDI_2022. Data and code for remaining microbiota analyses can be found at https://github.com/barronmr/antip40_microbiota. Raw 16S rRNA sequences have been deposited in the NCBI Sequence Repository Archive under the accession number PRJNA811422.

## References

[1] Britton RA, Young VB. 2012. Interaction between the intestinal microbiota and host in *Clostridum difficile* colonization resistance. Trends Microbiol 20:313–319. doi:10.1016/j.tim.2012.04.001. 22595318 PMC3408078

[2] Pike CM, Theriot CM. 2021. Mechanisms of colonization resistance against *Clostridioides difficile*. J Infect Dis 223:S194–S200. doi:10.1093/infdis/jiaa408. 33326565 PMC8206795

[3] Theriot CM, Koenigsknecht MJ, Carlson PE, Jr., Hatton GE, Nelson AM, Li B, Huffnagle GB, Li JZ, Young VB. 2014. Antibiotic-induced shifts in the mouse gut microbiome and metabolome increase susceptibility to *Clostridium difficile* infection. Nat Commun 5:3114. doi:10.1038/ncomms4114. 24445449 PMC3950275

[4] Chen X, Katchar K, Goldsmith JD, Nanthakumar N, Cheknis A, Gerding DN, Kelly CP. 2008. A mouse model of *Clostridium difficile*-associated disease. Gastroenterology 135:1984–1992. doi:10.1053/j.gastro.2008.09.002. 18848941

[5] Lewis BB, Buffie CG, Carter RA, Leiner I, Toussaint NC, Miller LC, Gobourne A, Ling L, Pamer EG. 2015. Loss of microbiota-mediated colonization resistance to *Clostridium difficile* infection with oral vancomycin compared with metronidazole. J Infect Dis 212:1656–1665. doi:10.1093/infdis/jiv256. 25920320 PMC4621244

[6] Lawley TD, Clare S, Walker AW, Goulding D, Stabler RA, Croucher N, Mastroeni P, Scott P, Raisen C, Mottram L, Fairweather NF, Wren BW, Parkhill J, Dougan G. 2009. Antibiotic treatment of *Clostridium difficile* carrier mice triggers a supershedder state, spore-mediated transmission, and severe disease in immunocompromised hosts. Infect Immun 77:3661–3669. doi:10.1128/IAI.00558-09. 19564382 PMC2737984

[7] Dubberke ER, Reske KA, Seiler S, Hink T, Kwon JH, Burnham CA. 2015. Risk factors for acquisition and loss of *Clostridium difficile* colonization inhospitalized patients. Antimicrob Agents Chemother 59:4533–4543. doi:10.1128/AAC.00642-15. 25987626 PMC4505269

[8] Gerding DN, Young VB, Donskey CJ. 2020. *Clostridioides difficile* (formerly *Clostridium difficile*) infection, p 2933–2947. *In* Bennett JE, Dolin R, Blaser MJ (ed), Mandell, Douglas, and Bennett's principles and practice of infectious diseases, Ninth ed. Elsevier.

[9] Fletcher JR, Pike CM, Parsons RJ, Rivera AJ, Foley MH, McLaren MR, Montgomery SA, Theriot CM. 2021. *Clostridioides difficile* exploits toxin-mediated inflammation to alter the host nutritional landscape and exclude competitors from the gut microbiota. Nat Commun 12:462–462. doi:10.1038/s41467-020-20746-4. 33469019 PMC7815924

[10] Abernathy-Close L, Barron MR, George JM, Dieterle MG, Vendrov KC, Bergin IL, Young VB. 2021. Intestinal inflammation and altered gut microbiota associated with inflammatory bowel disease render mice susceptible to *Clostridioides difficile* colonization and infection. mBio 12:e0273320. doi:10.1128/mBio.02733-20. 34126769 PMC8262858

[11] Nagao-Kitamoto H, Leslie JL, Kitamoto S, Jin C, Thomsson KA, Gillilland MG, 3rd, Kuffa P, Goto Y, Jenq RR, Ishii C, Hirayama A, Seekatz AM, Martens EC, Eaton KA, Kao JY, Fukuda S, Higgins PDR, Karlsson NG, Young VB, Kamada N. 2020. Interleukin-22-mediated host glycosylation prevents *Clostridioides difficile* infection by modulating the metabolic activity of the gut microbiota. Nat Med 26:608–617. doi:10.1038/s41591-020-0764-0. 32066975 PMC7160049

[12] Sartor RB, Wu GD. 2017. Roles for intestinal bacteria, viruses, and fungi in pathogenesis of inflammatory bowel diseases and therapeutic approaches. Gastroenterology 152:327–339. doi:10.1053/j.gastro.2016.10.012. 27769810 PMC5511756

[13] Ni J, Wu GD, Albenberg L, Tomov VT. 2017. Gut microbiota and IBD: causation or correlation? Nat Rev Gastroenterol Hepatol 14:573–584. doi:10.1038/nrgastro.2017.88. 28743984 PMC5880536

[14] Franzosa EA, Sirota-Madi A, Avila-Pacheco J, Fornelos N, Haiser HJ, Reinker S, Vatanen T, Hall AB, Mallick H, McIver LJ, Sauk JS, Wilson RG, Stevens BW, Scott JM, Pierce K, Deik AA, Bullock K, Imhann F, Porter JA, Zhernakova A, Fu J, Weersma RK, Wijmenga C, Clish CB, Vlamakis H, Huttenhower C, Xavier RJ. 2019. Gut microbiome structure and metabolic activity in inflammatory bowel disease. Nat Microbiol 4:293–305. doi:10.1038/s41564-018-0306-4. 30531976 PMC6342642

[15] Duboc H, Rajca S, Rainteau D, Benarous D, Maubert MA, Quervain E, Thomas G, Barbu V, Humbert L, Despras G, Bridonneau C, Dumetz F, Grill JP, Masliah J, Beaugerie L, Cosnes J, Chazouillères O, Poupon R, Wolf C, Mallet JM, Langella P, Trugnan G, Sokol H, Seksik P. 2013. Connecting dysbiosis, bile-acid dysmetabolism and gut inflammation in inflammatory bowel diseases. Gut 62:531–539. doi:10.1136/gutjnl-2012-302578. 22993202

[16] Wang W, Chen L, Zhou R, Wang X, Song L, Huang S, Wang G, Xia B, Forbes BA. 2014. Increased proportions of *Bifidobacterium* and the *Lactobacillus* group and loss of butyrate-producing bacteria in inflammatory bowel disease. J Clin Microbiol 52:398–406. doi:10.1128/JCM.01500-13. 24478468 PMC3911339

[17] Zhang T, Lin Q-Y, Fei J-X, Zhang Y, Lin M-Y, Jiang S-H, Wang P, Chen Y. 2016. *Clostridium difficile* infection worsen outcome of hospitalized patients with inflammatory bowel disease. Sci Rep 6:29791. doi:10.1038/srep29791. 27417996 PMC4945908

[18] Chen XL, Deng J, Chen X, Wan SS, Wang Y, Cao Q. 2019. High incidence and morbidity of *Clostridium difficile* infection among hospitalized patients with inflammatory bowel disease: a prospective observational cohort study. J Dig Dis 20:460–466. doi:10.1111/1751-2980.12798. 31278840

[19] Barber GE, Hendler S, Okafor P, Limsui D, Limketkai BN. 2018. Rising incidence of intestinal infections in inflammatory bowel disease: a nationwide analysis. Inflammatory Bowel Diseases 24:1849–1856. doi:10.1093/ibd/izy086. 29722832

[20] Rodemann JF, Dubberke ER, Reske KA, Seo DH, Stone CD. 2007. Incidence of *Clostridium difficile* infection in inflammatory bowel disease. Clin Gastroenterol Hepatol 5:339–344. doi:10.1016/j.cgh.2006.12.027. 17368233

[21] Ricciardi R, Ogilvie JW, Jr., Roberts PL, Marcello PW, Concannon TW, Baxter NN. 2009. Epidemiology of *Clostridium difficile* colitis in hospitalized patients with inflammatory bowel diseases. Dis Colon Rectum 52:40–45. doi:10.1007/DCR.0b013e31819733fd. 19273954

[22] Bossuyt P, Verhaegen J, Van Assche G, Rutgeerts P, Vermeire S. 2009. Increasing incidence of *Clostridium difficile*-associated diarrhea in inflammatory bowel disease. J Crohns Colitis 3:4–7. doi:10.1016/j.crohns.2008.09.003. 21172241

[23] Seekatz AM, Theriot CM, Molloy CT, Wozniak KL, Bergin IL, Young VB. 2015. Fecal microbiota transplantation eliminates *Clostridium difficile* in a murine model of relapsing disease. Infect Immun 83:3838–3846. doi:10.1128/IAI.00459-15. 26169276 PMC4567621

[24] Theriot CM, Koumpouras CC, Carlson PE, Bergin II, Aronoff DM, Young VB. 2011. Cefoperazone-treated mice as an experimental platform to assess differential virulence of *Clostridium difficile* strains. Gut Microbes 2:326–334. doi:10.4161/gmic.19142. 22198617 PMC3337121

[25] Cahill RJ, Foltz CJ, Fox JG, Dangler CA, Powrie F, Schauer DB. 1997. Inflammatory bowel disease: an immunity-mediated condition triggered by bacterial infection with *Helicobacter hepaticus*. Infect Immun 65:3126–3131. doi:10.1128/iai.65.8.3126-3131.1997. 9234764 PMC175441

[26] Kullberg MC, Rothfuchs AG, Jankovic D, Caspar P, Wynn TA, Gorelick PL, Cheever AW, Sher A. 2001. *Helicobacter hepaticus*-induced colitis in interleukin-10-deficient mice: cytokine requirements for the induction and maintenance of intestinal inflammation. Infect Immun 69:4232–4241. doi:10.1128/IAI.69.7.4232-4241.2001. 11401959 PMC98456

[27] Kullberg MC, Jankovic D, Feng CG, Hue S, Gorelick PL, McKenzie BS, Cua DJ, Powrie F, Cheever AW, Maloy KJ, Sher A. 2006. IL-23 plays a key role in *Helicobacter hepaticus*-induced T cell-dependent colitis. J Exp Med 203:2485–2494. doi:10.1084/jem.20061082. 17030948 PMC2118119

[28] Maloy KJ, Salaun L, Cahill R, Dougan G, Saunders NJ, Powrie F. 2003. CD4+CD25+ T(R) cells suppress innate immune pathology through cytokine-dependent mechanisms. J Exp Med 197:111–119. doi:10.1084/jem.20021345. 12515818 PMC2193798

[29] Britton RA, Young VB. 2014. Role of the intestinal microbiota in resistance to colonization by *Clostridium difficile*. Gastroenterology 146:1547–1553. doi:10.1053/j.gastro.2014.01.059. 24503131 PMC3995857

[30] Holmes I, Harris K, Quince C. 2012. Dirichlet multinomial mixtures: generative models for microbial metagenomics. PLoS One 7:e30126. doi:10.1371/journal.pone.0030126. 22319561 PMC3272020

[31] Segata N, Izard J, Waldron L, Gevers D, Miropolsky L, Garrett WS, Huttenhower C. 2011. Metagenomic biomarker discovery and explanation. Genome Biol 12:R60. doi:10.1186/gb-2011-12-6-r60. 21702898 PMC3218848

[32] Altmann A, Toloşi L, Sander O, Lengauer T. 2010. Permutation importance: a corrected feature importance measure. Bioinformatics 26:1340–1347. doi:10.1093/bioinformatics/btq134. 20385727

[33] Topçuoğlu BD, Lesniak NA, Ruffin MT, Wiens J, Schloss PD, Blaser MJ. 2020. A framework for effective application of machine learning to microbiome-based classification problems. mBio 11:e00434-20. doi:10.1128/mBio.00434-20. 32518182 PMC7373189

[34] Lawley TD, Young VB. 2013. Murine models to study *Clostridium difficile* infection and transmission. Anaerobe 24:94–97. doi:10.1016/j.anaerobe.2013.09.008. 24076318 PMC4089867

[35] Reeves AE, Theriot CM, Bergin IL, Huffnagle GB, Schloss PD, Young VB. 2011. The interplay between microbiome dynamics and pathogen dynamics in a murine model of *Clostridium difficile* infection. Gut Microbes 2:145–158. doi:10.4161/gmic.2.3.16333. 21804357 PMC3225775

[36] Schubert AM, Rogers MAM, Ring C, Mogle J, Petrosino JP, Young VB, Aronoff DM, Schloss PD, Fraser CM. 2014. Microbiome data distinguish patients with *Clostridium difficile* infection and non-*C. difficile*-associated diarrhea from healthy controls. mBio 5:e01021-14. doi:10.1128/mBio.01021-14. 24803517 PMC4010826

[37] Gu S, Chen Y, Zhang X, Lu H, Lv T, Shen P, Lv L, Zheng B, Jiang X, Li L. 2016. Identification of key taxa that favor intestinal colonization of *Clostridium difficile* in an adult Chinese population. Microbes Infect 18:30–38. doi:10.1016/j.micinf.2015.09.008. 26383014

[38] Reeves AE, Koenigsknecht MJ, Bergin IL, Young VB, McCormick BA. 2012. Suppression of *Clostridium difficile* in the gastrointestinal tracts of germfree mice inoculated with a murine isolate from the family *Lachnospiraceae*. Infect Immun 80:3786–3794. doi:10.1128/IAI.00647-12. 22890996 PMC3486043

[39] Lesniak NA, Schubert AM, Sinani H, Schloss PD, Britton RA. 2021. Clearance of *Clostridioides difficile* colonization is associated with antibiotic-specific bacterial changes. mSphere 6:e01238-20. doi:10.1128/mSphere.01238-20. 33952668 PMC8103992

[40] Schubert AM, Sinani H, Schloss PD, Fraser CM. 2015. Antibiotic-induced alterations of the murine gut microbiota and subsequent effects on colonization resistance against *Clostridium difficile*. mBio 6:e00974-15. doi:10.1128/mBio.00974-15. 26173701 PMC4502226

[41] Lee YJ, Arguello ES, Jenq RR, Littmann E, Kim GJ, Miller LC, Ling L, Figueroa C, Robilotti E, Perales M-A, Barker JN, Giralt S, van den Brink MRM, Pamer EG, Taur Y. 2017. Protective factors in the intestinal microbiome against *Clostridium difficile* infection in recipients of allogeneic hematopoietic stem cell transplantation. J Infect Dis 215:1117–1123. doi:10.1093/infdis/jix011. 28498996 PMC5426375

[42] Gregory AL, Pensinger DA, Hryckowian AJ. 2021. A short chain fatty acid–centric view of *Clostridioides difficile* pathogenesis. PLoS Pathog 17:e1009959. doi:10.1371/journal.ppat.1009959. 34673840 PMC8530303

[43] Antharam VC, Li EC, Ishmael A, Sharma A, Mai V, Rand KH, Wang GP. 2013. Intestinal dysbiosis and depletion of butyrogenic bacteria in *Clostridium difficile* infection and nosocomial diarrhea. J Clin Microbiol 51:2884–2892. doi:10.1128/JCM.00845-13. 23804381 PMC3754663

[44] Seekatz AM, Theriot CM, Rao K, Chang Y-M, Freeman AE, Kao JY, Young VB. 2018. Restoration of short chain fatty acid and bile acid metabolism following fecal microbiota transplantation in patients with recurrent *Clostridium difficile* infection. Anaerobe 53:64–73. doi:10.1016/j.anaerobe.2018.04.001. 29654837 PMC6185828

[45] Kakiyama G, Pandak WM, Gillevet PM, Hylemon PB, Heuman DM, Daita K, Takei H, Muto A, Nittono H, Ridlon JM, White MB, Noble NA, Monteith P, Fuchs M, Thacker LR, Sikaroodi M, Bajaj JS. 2013. Modulation of the fecal bile acid profile by gut microbiota in cirrhosis. J Hepatol 58:949–955. doi:10.1016/j.jhep.2013.01.003. 23333527 PMC3936319

[46] Theriot CM, Bowman AA, Young VB, Ellermeier CD. 2016. Antibiotic-induced alterations of the gut microbiota alter secondary bile acid production and allow for *Clostridium difficile* spore germination and outgrowth in the large intestine. mSphere 1:e00045-15. doi:10.1128/mSphere.00045-15. PMC4863611 27239562

[47] Hryckowian AJ, Van Treuren W, Smits SA, Davis NM, Gardner JO, Bouley DM, Sonnenburg JL. 2018. Microbiota-accessible carbohydrates suppress *Clostridium difficile* infection in a murine model. Nat Microbiol 3:662–669. doi:10.1038/s41564-018-0150-6. 29686297 PMC6126909

[48] Guinan J, Wang S, Hazbun TR, Yadav H, Thangamani S. 2019. Antibiotic-induced decreases in the levels of microbial-derived short-chain fatty acids correlate with increased gastrointestinal colonization of *Candida albicans*. Sci Rep 9:8872. doi:10.1038/s41598-019-45467-7. 31222159 PMC6586901

[49] Hove H, Tvede M, Mortensen PB. 1996. Antibiotic-associated diarrhoea, *Clostridium difficile*, and short-chain fatty acids. Scand J Gastroenterol 31:688–693. doi:10.3109/00365529609009151. 8819219

[50] Aguirre AM, Yalcinkaya N, Wu Q, Swennes A, Tessier ME, Roberts P, Miyajima F, Savidge T, Sorg JA. 2021. Bile acid-independent protection against Clostridioides difficile infection. PLoS Pathog 17:e1010015. doi:10.1371/journal.ppat.1010015. 34665847 PMC8555850

[51] Barron MR, Young VB, Bucci V. 2022. Viewing bacterial colonization through the lens of systems biology. mSystems 7:e01383-21. doi:10.1128/msystems.01383-21. PMC9040567 35354321

[52] Khan I, Ullah N, Zha L, Bai Y, Khan A, Zhao T, Che T, Zhang C. 2019. Alteration of gut microbiota in inflammatory bowel disease (IBD): cause or consequence? IBD treatment targeting the gut microbiome. Pathogens (Basel, Switzerland) 8:126. doi:10.3390/pathogens8030126. PMC6789542 31412603

[53] Yilmaz B, Juillerat P, Øyås O, Ramon C, Bravo FD, Franc Y, Fournier N, Michetti P, Mueller C, Geuking M, Pittet VEH, Maillard MH, Rogler G, Abdelrahman K, Ademi G, Aepli P, Thomas A, Anderegg C, Antonino A-T, Archanioti E, Arrigoni E, Bakker de JD, Balsiger B, Bastürk P, Bauerfeind P, Becocci A, Belli D, Bengoa JM, Biedermann L, Binek J, Blattmann M, Boehm S, Boldanova T, Borovicka J, Braegger CP, Brand S, Brügger L, Brunner S, Bühr P, Burnand B, Burk S, Burri E, Buyse S, Cao D-T, Carstens O, Criblez DH, Cunningham S, D’Angelo F, de Saussure P, Degen L, Swiss IBD Cohort Investigators, et al. 2019. Microbial network disturbances in relapsing refractory Crohn’s disease. Nat Med 25:323–336. doi:10.1038/s41591-018-0308-z. 30664783

[54] Perez J, Springthorpe VS, Sattar SA. 2011. Clospore: a liquid medium for producing high titers of semi-purified spores of *Clostridium difficile*. J AOAC Int 94:618–626. doi:10.1093/jaoac/94.2.618. 21563698

[55] Abernathy-Close L, Dieterle MG, Vendrov KC, Bergin IL, Rao K, Young VB. 2020. Aging dampens the intestinal innate immune response during severe *Clostridioides difficile* infection and is associated with altered cytokine levels and granulocyte mobilization. Infect Immun 88:e00960-19. doi:10.1128/IAI.00960-19. 32284366 PMC7240091

[56] Pratt JS, Sachen KL, Wood HD, Eaton KA, Young VB. 2006. Modulation of host immune responses by the cytolethal distending toxin of *Helicobacter hepaticus*. Infect Immun 74:4496–4504. doi:10.1128/IAI.00503-06. 16861635 PMC1539592

[57] Young VB, Knox KA, Pratt JS, Cortez JS, Mansfield LS, Rogers AB, Fox JG, Schauer DB. 2004. In vitro and in vivo characterization of *Helicobacter hepaticus* cytolethal distending toxin mutants. Infect Immun 72:2521–2527. doi:10.1128/IAI.72.5.2521-2527.2004. 15102759 PMC387909

[58] Warren CA, van Opstal E, Ballard TE, Kennedy A, Wang X, Riggins M, Olekhnovich I, Warthan M, Kolling GL, Guerrant RL, Macdonald TL, Hoffman PS. 2012. Amixicile, a novel inhibitor of pyruvate: ferredoxin oxidoreductase, shows efficacy against *Clostridium difficile* in a mouse infection model. Antimicrob Agents Chemother 56:4103–4111. doi:10.1128/AAC.00360-12. 22585229 PMC3421617

[59] Nagalingam NA, Robinson CJ, Bergin IL, Eaton KA, Huffnagle GB, Young VB. 2013. The effects of intestinal microbial community structure on disease manifestation in IL-10−/− mice infected with *Helicobacter hepaticus*. Microbiome 1:15. doi:10.1186/2049-2618-1-15. 24450737 PMC3971628

[60] Koenigsknecht MJ, Theriot CM, Bergin IL, Schumacher CA, Schloss PD, Young VB. 2015. Dynamics and establishment of *Clostridium difficile* infection in the murine gastrointestinal tract. Infect Immun 83:934–941. doi:10.1128/IAI.02768-14. 25534943 PMC4333439

[61] Kozich JJ, Westcott SL, Baxter NT, Highlander SK, Schloss PD. 2013. Development of a dual-index sequencing strategy and curation pipeline for analyzing amplicon sequence data on the MiSeq Illumina sequencing platform. Appl Environ Microbiol 79:5112–5120. doi:10.1128/AEM.01043-13. 23793624 PMC3753973

[62] Schloss PD, Westcott SL, Ryabin T, Hall JR, Hartmann M, Hollister EB, Lesniewski RA, Oakley BB, Parks DH, Robinson CJ, Sahl JW, Stres B, Thallinger GG, Van Horn DJ, Weber CF. 2009. Introducing mothur: open-source, platform-independent, community-supported software for describing and comparing microbial communities. Appl Environ Microbiol 75:7537–7541. doi:10.1128/AEM.01541-09. 19801464 PMC2786419

[63] Edgar RC, Haas BJ, Clemente JC, Quince C, Knight R. 2011. UCHIME improves sensitivity and speed of chimera detection. Bioinformatics Bioinformatics 27:2194–2200. doi:10.1093/bioinformatics/btr381. 21700674 PMC3150044

[64] Westcott SL, Schloss PD. 2017. OptiClust, an improved method for assigning amplicon-based sequence data to operational taxonomic units. mSphere 2:e00073-17. doi:10.1128/mSphereDirect.00073-17. 28289728 PMC5343174

[65] Topçuoğlu BD, Lapp Z, Sovacool KL, Snitkin E, Wiens J, Schloss PD. 2021. mikropml: user-friendly R package for supervised machine learning pipelines. J Open Source Softw 6:3073. doi:10.21105/joss.03073. 34414351 PMC8372219

[66] Saito T, Rehmsmeier M. 2015. The precision-recall plot is more informative than the ROC plot when evaluating binary classifiers on imbalanced datasets. PLoS One 10:e0118432. doi:10.1371/journal.pone.0118432. 25738806 PMC4349800

