# Supplementary figures and images for: Intestinal Inflammation Reversibly Alters the Microbiota to Drive Susceptibility to Clostridioides difficile Colonization in a Mouse Model of Colitis
Source: mBio. 2022 Jul 28;13(4):e01904-22. doi: 10.1128/mbio.01904-22 (PMC9426610; doi:10.1128/mbio.01904-22)

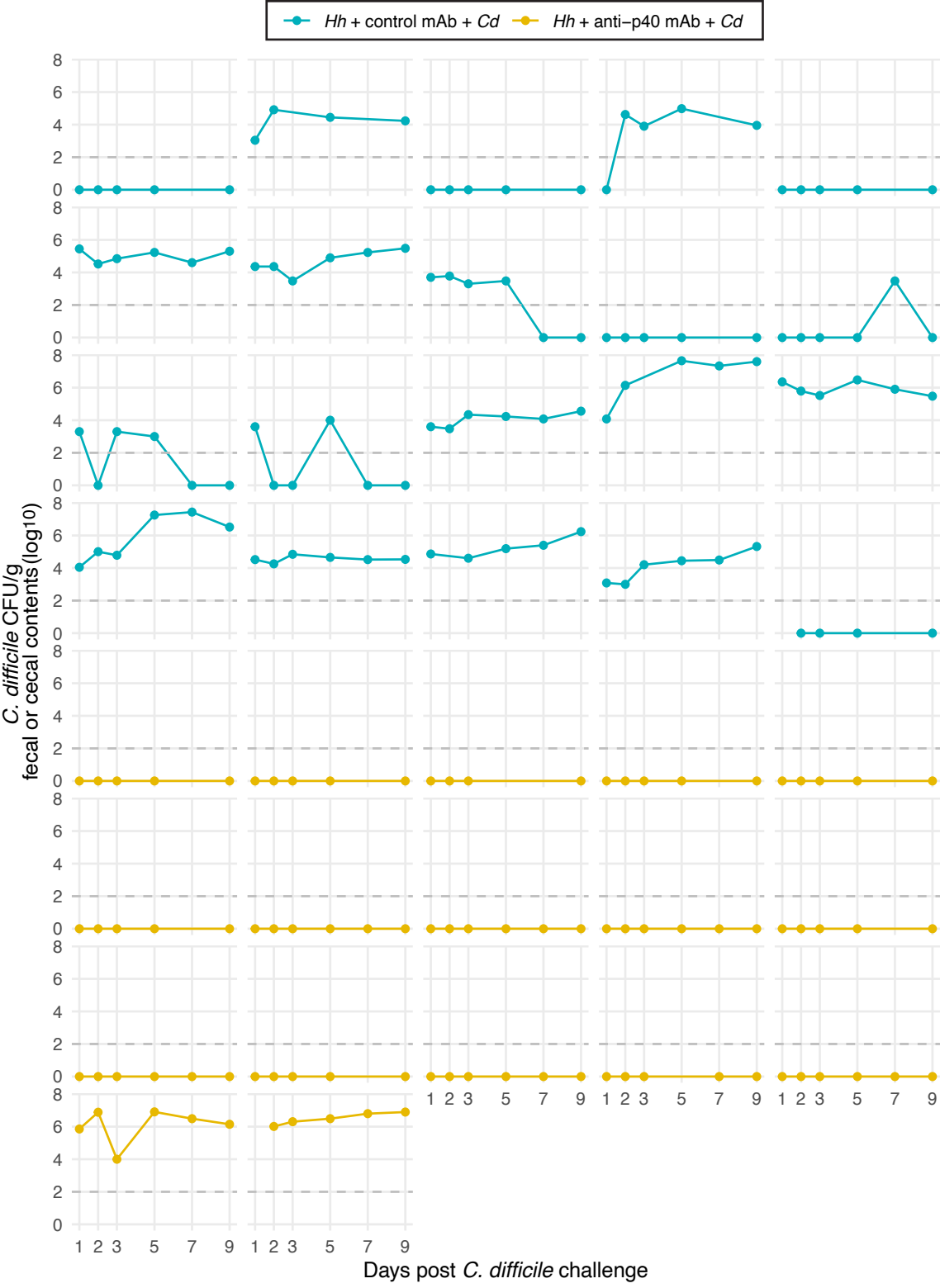

Supplement: FIG S2 [file mbio.01904-22-s0002.pdf]

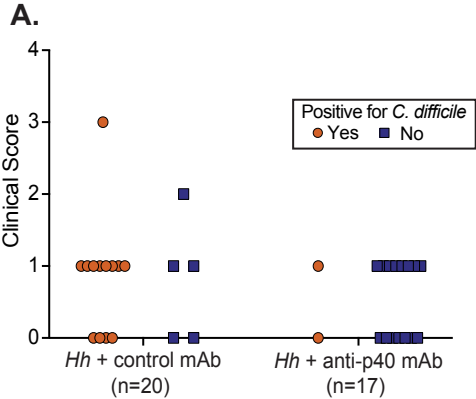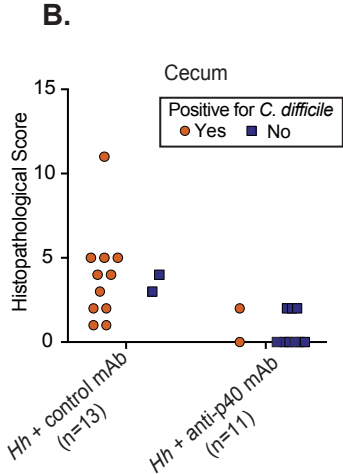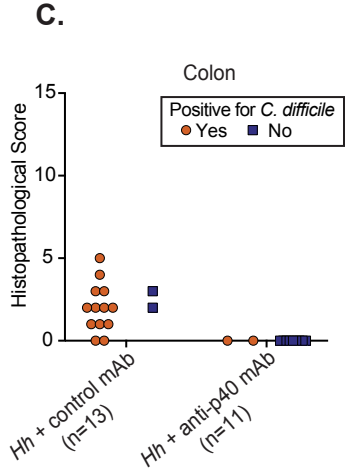

Supplement: FIG S3 [file mbio.01904-22-s0003.pdf]

Enterotype

1 2 3

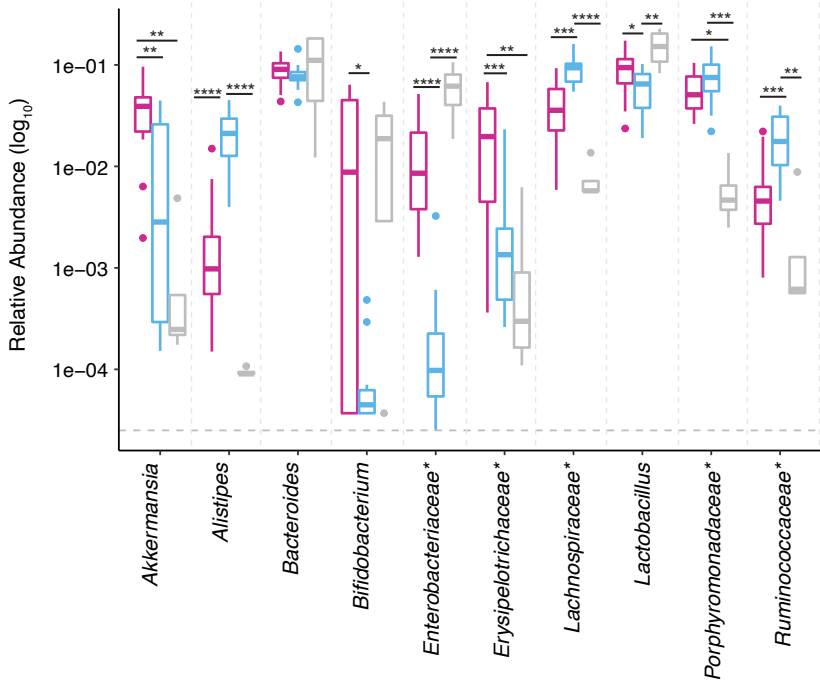

Supplement: FIG S5 [file mbio.01904-22-s0005.pdf]

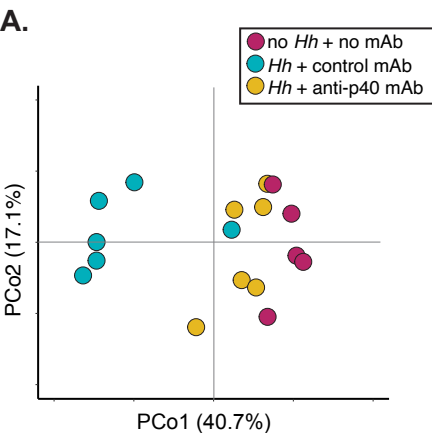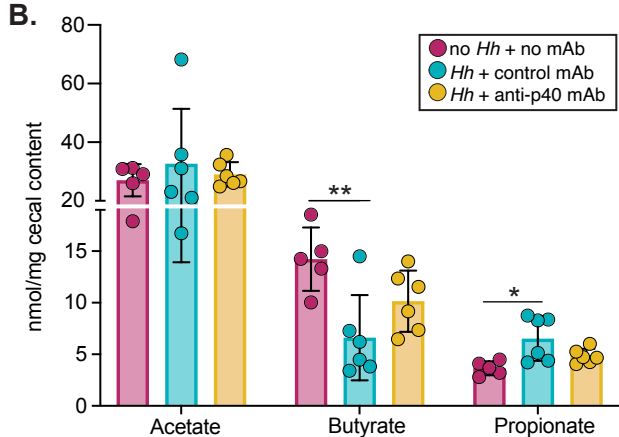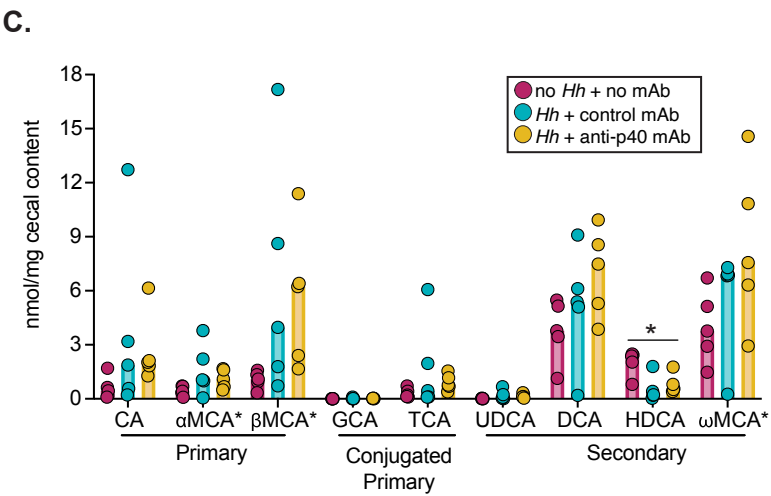

Supplement: FIG S4 [file mbio.01904-22-s0004.pdf]
