# Supplementary material for: Intestinal Inflammation Reversibly Alters the Microbiota to Drive Susceptibility to Clostridioides difficile Colonization in a Mouse Model of Colitis
Source: mBio. 2022 Jul 28;13(4):e01904-22. doi: 10.1128/mbio.01904-22 (PMC9426610; doi:10.1128/mbio.01904-22)
Supplement: TABLE S2 [file mbio.01904-22-s0008.pdf]

Table S2 Feature Importance analyses, top 20 OTUs (corresponds with Fig. 4D in main text).

OTU	mean decrease in AUROC	standard deviation	% models	rank
Enterobacteriaceae (OTU 7)	-0.0078371	0.0121111	58.0	1
Erysipelotrichaceae (OTU 222)	-0.0022672	0.0047096	86.4	2
Porphyromonodaceae (OTU 14)	-0.0022489	0.0041229	69.4	3
Erysipelotrichaceae (OTU 10)	-0.0017732	0.0045827	78.4	4
Ruminococcaceae (OTU 56)	-0.0015730	0.0047184	60.6	5
Lachnospiraceae (OTU 54)	-0.0015468	0.0051692	65.8	6
Lachnospiraceae (OTU 27)	-0.0012169	0.0046644	59.6	7
Bifidobacterium (OTU 12)	-0.0010722	0.0034798	75.0	8
Lachnnospiraceae (OTU 98)	-0.0009233	0.0031928	86.4	9
Firmicutes (OTU 69)	-0.0008873	0.0051307	77.6	10
Clostridium IV (OTU 126)	-0.0007600	0.0042812	45.2	11
Lachnospiraceae (OTU 23)	-0.0007161	0.0036369	60.4	12
Osciillibacter (OTU 164)	-0.0006656	0.0034880	78.2	13
Porphyromonodaceae (OTU 29)	-0.0006512	0.0032917	65.4	14
Lactobacillus (OTU 26)	-0.0006454	0.0030530	85.2	15
Acetatifactor (OTU 172)	-0.0006135	0.0026414	87.8	16
Lactobacillus (OTU 15)	-0.0005947	0.0026614	85.4	17
Dorea (OTU 149)	-0.0005938	0.0025554	84.8	18
Lachnospiraceae (OTU 38)	-0.0005561	0.0030798	85.6	19
Helicobacter (OTU 21)	-0.0005545	0.0023031	93.6	20
