# Supplementary material for: Intestinal Inflammation Reversibly Alters the Microbiota to Drive Susceptibility to Clostridioides difficile Colonization in a Mouse Model of Colitis
Source: mBio. 2022 Jul 28;13(4):e01904-22. doi: 10.1128/mbio.01904-22 (PMC9426610; doi:10.1128/mbio.01904-22)
Supplement: TABLE S1 [file mbio.01904-22-s0007.pdf]

Table S1 Primers and probes used for PCR and RT-PCR analyses

**Target**	**Primer and Probe Sequences (5’-3’)**	**Reference**
H. hepaticus 16S rRNA	Forward: GCATTTGAAACTGTTACTCTG<br>Reverse: CTGTTTTCAAGCTCCCC	(1)
β-actin	Forward: CCGTGAAAAGATGACCCAGATC<br>Reverse: CACAGCCTGGATGGCTACGT<br>Probe: TTGAGACCTTCAACACCCCA	(2)
IL-17A	Forward: GGACTCTCCACCGCAATGAA<br>Reverse: CTCTCAGGCTCCCTCTTCAG<br>Probe: CTCAGTGCCGCCACCAGCGC	Bethany Moore’s lab; University of Michigan
TNF-α	Forward: CCAGACCCTCACACTCAGATCA<br>Reverse: CCTCCACTTGGTGGTTTGCT<br>Probe: TCGAGTGACAAGCCTGTAGCCCACG	(3)
IFN-γ	Forward: GCAACAGCAAGGCGAGAAA<br>Reverse: GCTGGATTCCGGCAACAG<br>Probe: AGGTCAACAACCCACAGGTCCAGCG’	(4)
IL-22	Forward: GACAGGTTCCAGCCCTACAT<br>Reverse: CTGGATGTTCTGGTCGTCAC<br>Probe: CAGGAAAGGCACCACCTCCTGC	(5)

References

1. Young VB, Knox KA, Pratt JS, Cortez JS, Mansfield LS, Rogers AB, Fox JG, Schauer DB. 2004. *In vitro* and i*n vivo* characterization of *Helicobacter hepaticus* cytolethal distending toxin mutants. Infection and Immunity 72:2521-2527.

2. Martínez-Colón GJ, Warheit-Niemi H, Gurczynski SJ, Taylor QM, Wilke CA, Podsiad AB, Crespo J, Bhan U, Moore BB. 2019. Influenza-induced immune suppression to methicillin-resistant *Staphylococcus aureus* is mediated by TLR9. PLoS pathogens 15:e1007560-e1007560.

3. Gurczynski SJ, Procario MC, O'Dwyer DN, Wilke CA, Moore BB. 2016. Loss of CCR2 signaling alters leukocyte recruitment and exacerbates γ-herpesvirus-induced pneumonitis and fibrosis following bone marrow transplantation. American Journal of Physiology Lung Cellular and Molecular Physiology 311:L611-L627.

4. Zhou X, Loomis-King H, Gurczynski SJ, Wilke CA, Konopka KE, Ptaschinski C, Coomes SM, Iwakura Y, van Dyk LF, Lukacs NW, Moore BB. 2016. Bone marrow transplantation alters lung antigen-presenting cells to promote TH17 response and the development of pneumonitis and fibrosis following gammaherpesvirus infection. Mucosal Immunology 9:610-620.

5. Xue J, Habtezion A. 2014. Carbon monoxide-based therapy ameliorates acute pancreatitis via TLR4 inhibition. The Journal of Clinical Investigation 124:437-447.
