# Supplementary material for: Intestinal Inflammation Reversibly Alters the Microbiota to Drive Susceptibility to Clostridioides difficile Colonization in a Mouse Model of Colitis
Source: mBio. 2022 Jul 28;13(4):e01904-22. doi: 10.1128/mbio.01904-22 (PMC9426610; doi:10.1128/mbio.01904-22)
Supplement: FIG S6 [file mbio.01904-22-s0006.pdf]

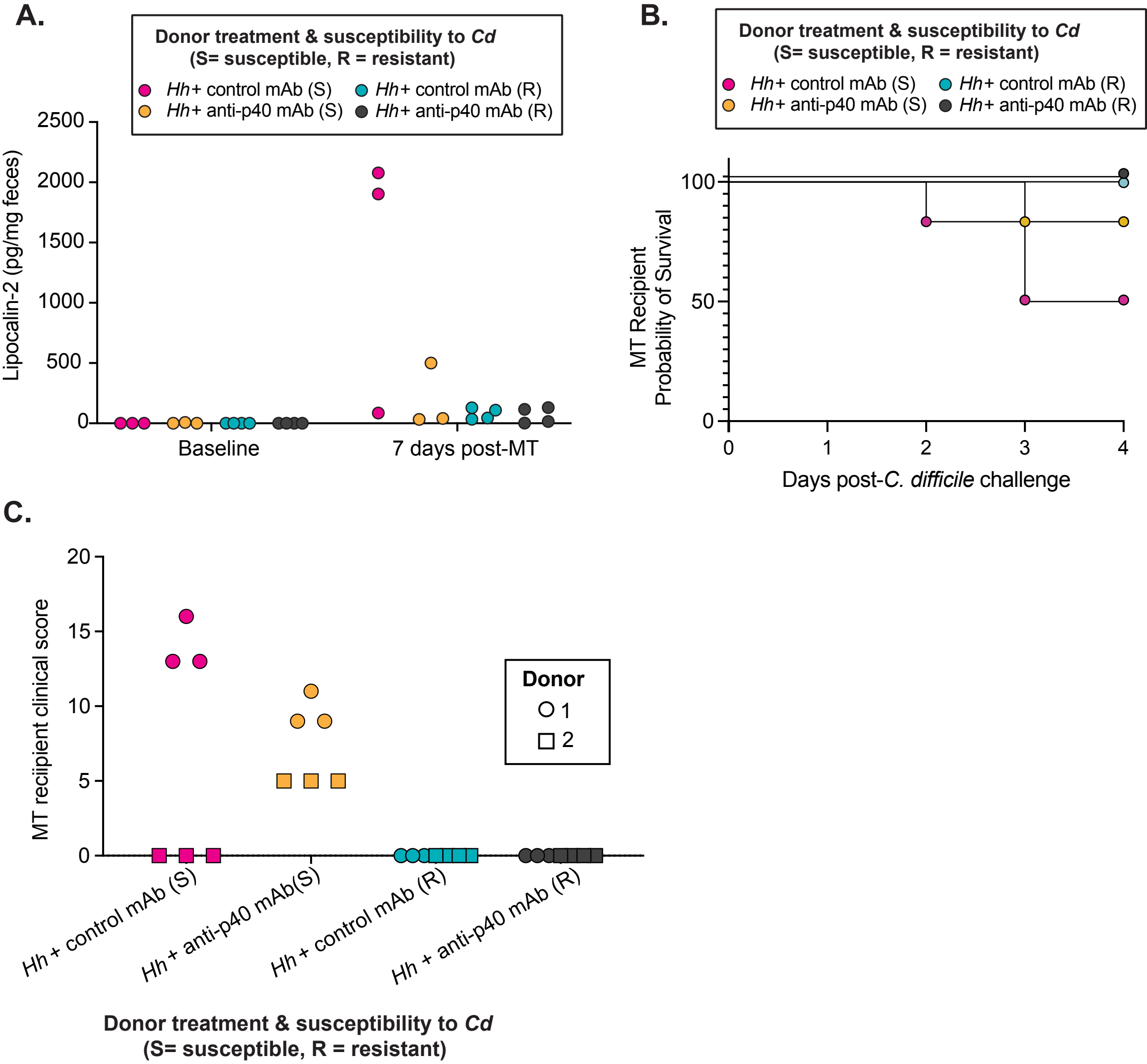
A.
Donor treatment & susceptibility to Cd
(S= susceptible, R = resistant)
Hh+ control mAb (S)
Hh+ control mAb (R)
Hh+ anti-p40 mAb (S)
Hh+ anti-p40 mAb (R)
Lipocalin-2 (pg/mg feces)
2500
2000
1500
1000
500
0
Baseline
7 days post-MT
B.
Donor treatment & susceptibility to Cd
(S= susceptible, R = resistant)
Hh+ control mAb (S)
Hh+ control mAb (R)
Hh+ anti-p40 mAb (S)
Hh+ anti-p40 mAb (R)
MT Recipient
Probability of Survival
100
50
0
0
1
2
3
4
Days post-C. difficile challenge
C.
MT recipient clinical score
20
15
10
5
0
Donor
1
2
Hh + control mAb (S)
Hh + anti-p40 mAb(S)
Hh + control mAb (R)
Hh + anti-p40 mAb (R)
Donor treatment & susceptibility to Cd
(S= susceptible, R = resistant)
